# Genetic Diversity and Population Structure in a Legacy Collection of Spring Barley Landraces Adapted to a Wide Range of Climates

**DOI:** 10.1371/journal.pone.0116164

**Published:** 2014-12-26

**Authors:** Raj K. Pasam, Rajiv Sharma, Alexander Walther, Hakan Özkan, Andreas Graner, Benjamin Kilian

**Affiliations:** 1 Department of Genebank, Leibniz-Institute of Plant Genetics and Crop Plant Research (IPK), Corrensstrasse 3, 06466 Gatersleben, Germany; 2 Department of Earth Sciences, University of Gothenburg, SE-405 30 Göteborg, Sweden; 3 Department of Field Crops, Faculty of Agriculture, University of Cukurova, 01330 Adana, Turkey; Nanjing Agricultural University, China

## Abstract

Global environmental change and increasing human population emphasize the urgent need for higher yielding and better adapted crop plants. One strategy to achieve this aim is to exploit the wealth of so called landraces of crop species, representing diverse traditional domesticated populations of locally adapted genotypes. In this study, we investigated a comprehensive set of 1485 spring barley landraces (Lrc1485) adapted to a wide range of climates, which were selected from one of the largest genebanks worldwide. The landraces originated from 5° to 62.5° N and 16° to 71° E. The whole collection was genotyped using 42 SSR markers to assess the genetic diversity and population structure. With an average allelic richness of 5.74 and 372 alleles, Lrc1485 harbours considerably more genetic diversity than the most polymorphic current GWAS panel for barley. Ten major clusters defined most of the population structure based on geographical origin, row type of the ear and caryopsis type – and were assigned to specific climate zones. The legacy core reference set Lrc648 established in this study will provide a long-lasting resource and a very valuable tool for the scientific community. Lrc648 is best suited for multi-environmental field testing to identify candidate genes underlying quantitative traits but also for allele mining approaches.

## Introduction

Cultivated barley, *Hordeum vulgare* L. ssp. *vulgare*, the domesticated form of wild barley *H. vulgare* L. ssp. *spontaneum* (C. Koch) Thell., is one of the oldest cereal crops [1]. Barley withstands hot and dry climates, marginal soils, to some extent salinity and a broad range of soil pH conditions [2], [3]. Today, barley is grown from 70° N in Norway to 46° S in Chile. The morphological, physiological and functional variation in barley reflects the underlying genetic diversity, which eases the environmental adaptation of this species [4], [5].

In barley, as in most crops, genetic bottlenecks occurred during domestication and crop improvement. For most loci current elite varieties harbour less genetic diversity than their wild relatives or early domesticates [6]–[9]. Landraces are traditional domesticated populations of locally adapted genotypes maintained by local farmers over generations. Early barley cultivars were direct selections among landraces or descended from genetic recombination of different landraces. Since then, new barley varieties are mainly developed through the reshuffling of alleles resulting in a more or less constant repertoire of alleles within the elite gene pool. Overall, the genetic basis in present elite barley breeding materials is rather limited.

Since the beginning of the 20^th^-century landraces were largely replaced by modern cultivars [10], [11], which are higher yielding under optimal conditions but which can completely fail under harsh environments [12]. Today, most barley landraces have disappeared from practical farming. Many of them are still maintained in *ex situ* repositories. Globally, landraces represent the largest part of barley germplasm conserved in genebanks (44%, 128.870 accessions) [13], [14].

Examples demonstrating the utilization of landrace genetic diversity include the introgression of i) plant height dwarfing alleles (*Rht1* and *Rht2*) derived from the Japanese wheat landrace “Shiro Daruma” [15], ii) several disease and insect resistance genes in wheat [16], [17], iii) submergence tolerance (*Sub1*) in rice [18], iv) broad spectrum powdery mildew resistance allele *mlo11* (from an Ethiopian barley landrace) [19], the rym4 virus resistance [20] or v) the boron-toxicity tolerance in barley, which was obtained from the Algerian landrace “Sahara” [21].

Genome-wide association analysis (GWAS), a population based method to identify marker-trait associations based on linkage disequilibrium (LD) is being recently used extensively in crop plants [22]. Genetic diversity, population size and stratification, extent of genome-wide LD, allele frequency distribution, marker type and coverage as well as other parameters determine the accuracy, resolution and power of GWAS [22]–[24]. As a consequence of genetic bottlenecks during domestication and crop improvement, allele frequency changes resulted in different levels of LD and genetic diversity. Thus, the extent of LD increases from wild barley to landrace and to elite cultivars, whereas the reverse trend was observed for genetic diversity [25]. The trade-off of higher LD in current cultivars is lower resolution in GWAS [26]. To fine-map QTL, the varying extent of LD observed in different genepools of barley (e.g. wild, landraces, and cultivars) could be exploited and provides a great opportunity for high-resolution association mapping [27].

Several studies were performed in barley to investigate genetic diversity in different germplasm collections using molecular markers, and few of these collections (panels) were established for GWAS. However, most studies were based on either cultivar collections [28]–[30], or mixtures of cultivars and landraces [31]–[33] or landrace panels from distinct geographical regions only [34]–[37]. Very few collections of barley landraces collected from a wider geographic range were established but none of them was designed for higher resolution GWAS [11], [38]–[39].Here we describe the establishment of a comprehensive and diverse collection of spring barley landraces adapted to a wide range of climates (Landrace Collection 1485, “Lrc1485”). We studied genetic diversity and population structure using microsatellite (Simple Sequence Repeat, SSR) markers, which provided the basis for targeted research activities. The legacy core reference set (CRS) Lrc648 established here is available for the scientific community to integrate data and to improve our elite barley varieties under changing environmental conditions.

## Material and Methods

### Plant material

**Figure 1:**
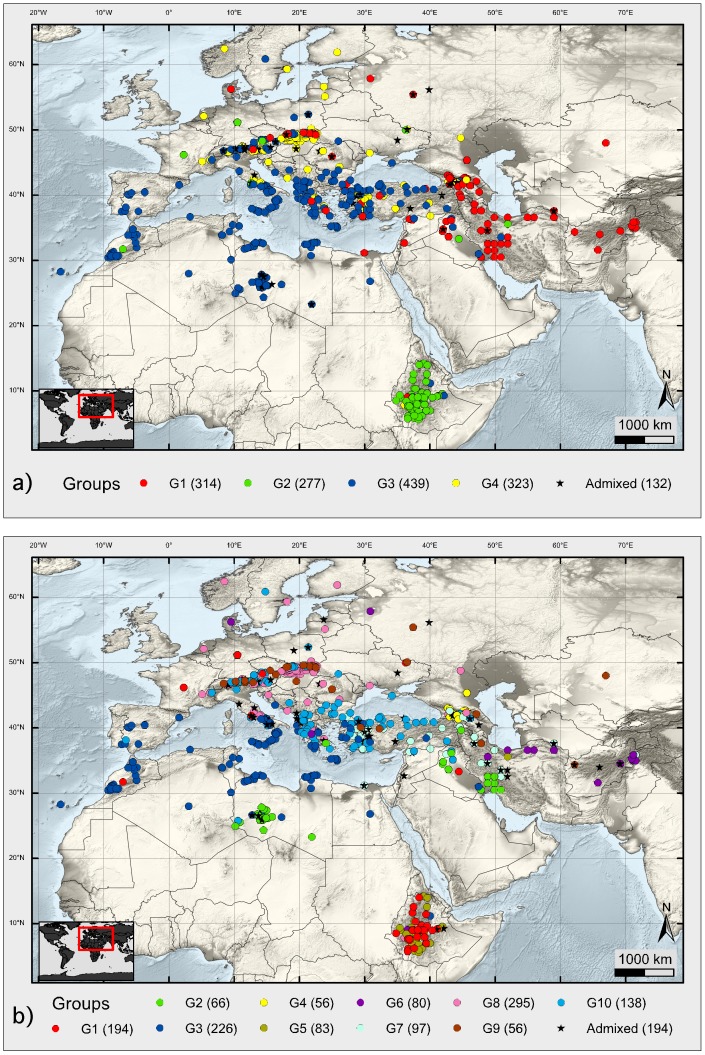
Geographic information system (GIS)-based topographic maps indicate the natural distribution of 1485 barley landrace accessions. (a) Structure (*K* = 4); (b) Structure (*K* = 10) - inferred clusters. Every individual accession is represented by a coloured circle indicating the membership to a cluster. Admixed accessions are indicated by black stars. See Fig. 2, Fig. 3, S2 Fig., S3 Fig., S4 Fig., Table 1, Table 3, Table 4, S3 Table and S1 Table for more information.

In total, 1491 spring barley landrace accessions adapted to diverse climate conditions were originally considered for this study. This representative collection was carefully selected among 22,093 *Hordeum* accessions available in 2008 at the “Federal *ex-situ* Genebank for Agricultural and Horticultural Crop Plants” maintained at the Leibniz Institute of Plant Genetics and Crop Plant Research (IPK) in Gatersleben, Germany. The landraces originated from 41 countries in Europe, West and Central Asia, North and East Africa and covering 5.63°–62.47° N and 16.62°–71.5° E. The selection was based on the following criteria: i) Mansfeld's taxonomical classification system considering growth habit, row type of the ear, kernel coverage, spike density, and seed colour [40], ii) the collection site had to be well documented (S1 Table), and iii) passport data (Characterization and Evaluation data since 1946) - obtained from IPK's Genebank Information System, GBIS, http://gbis.ipk-gatersleben.de). Barley landraces collected during targeted expeditions before 1992 were considered whenever possible to work with trustable materials. From Syria and Jordan only a few landraces (six accessions) were included - as barley landraces from this region were comprehensively investigated by [34], [41]. Apart from other considerations, the proportion of landraces selected from each country should represent the overall composition of IPK's spring barley landrace collection containing 6,800 accessions meaning the number of accessions per country differed (Fig. 1, Table 1, S1 Table).

**Table 1:** Distribution of landraces considered in this study according to their countries of origin, caryopsis type and row type.

		Hulled		Naked	
Country of origin	No. of accessions	Two- rowed	Six- rowed	Two- rowed	Six- rowed
Afghanistan	107	9	87	1	10
Albania	22	5	10	2	5
Algeria	5	2	3	0	0
Armenia	4	4	0	0	0
Austria	56	36	20	0	0
Azerbaijan	1	0	1	0	0
Bulgaria	15	0	14	0	1
Croatia	3	2	1	0	0
Czech	17	7	1	7	2
Denmark	2	0	0	0	2
Egypt	5	1	4	0	0
Ethiopia	299	54	46	95	104
Finland	3	3	0	0	0
France	9	2	6	0	1
Georgia	80	47	33	0	0
Germany	37 (1)	26	7	3 (1)	1
Greece	70 (1)	19	50 (1)	0	1
Hungary	3	2	0	0	1
Iran	84	44	31	9	0
Iraq	37 (1)	14 (1)	22	1	0
Italy	42	9	28	0	5
Kazakhstan	1	0	0	1	0
Latvia	1	1	0	0	0
Libya	123	13	107	0	3
Lithuania	1	1	0	0	0
Macedonia	1	0	1	0	0
Morocco	50	1	48	0	1
Netherlands	1	1	0	0	0
Norway	1	1	0	0	0
Poland	58	40	8	10	0
Romania	10	5	3	1	1
Russia	23 (1)	5	3	12 (1)	3
Slovakia	149 (2)	146 (2)	3	0	0
Spain	34	0	34	0	0
Sweden	3	2	1	0	0
Switzerland	10	8	1	0	1
Syria	6	5	1	0	0
Tunisia	4	1	3	0	0
Turkey	99	48	50	1	0
Ukraine	6	2	3	0	1
Yugoslavia*	9	4	5	0	0
Total Considered	1491 (6) 1485	570 (3) 567	635 (1) 634	143 (2) 141	143 (0) 143

*Former Yugoslavia (incl. Serbia, Kosovo, Bosnia and Herzegovina). Numbers in brackets indicate the number of accessions excluded from analyses. Thus in total 1485 accessions were considered for analyses.

The IPK genebank practices the splitting of original landrace accessions and maintains them as morphologically distinct accessions to counteract the possible loss of rare alleles in the original population of genotypes [42], [43]. For this reason, a current landrace accession might correspond to a single representative genotype of the original landrace population collected. Whenever possible, latitude and longitude coordinates were inferred using the original collection site descriptions. A broader source location (nearby city or province or country capital) was considered to infer the geographic coordinates, when the exact collection location was not documented. Searches were performed using Google maps (http://maps.google.com/maps) and global Gazetteer version 2.2 (http://www.fallingrain.com/world/index.html).

### Genotyping using SSR markers

Four seeds per landrace accession were randomly selected and sown under greenhouse conditions at IPK in 2008. Leaf material from one representative plant per accession was harvested three weeks after sowing. DNA was isolated from freeze-dried leaves using a BioRobot 9600 Work Station and the MagAttract 96 DNA plant core kit (Qiagen, Germany). Forty-five fluorescence-labelled SSR markers were selected based on their mapping position in the barley genome, covering all seven chromosomes [44], [45] (Table 2, S1 Fig.). Primers were labelled with HEX, FAM and TAMRA dyes allowing multiplexing of primers pairs into 15 multiplexes (M1 to M15) with three primer pairs per amplification. PCR reactions were performed following the protocols described by [46]. Amplification products were separated on a MegaBACE 1000 capillary sequencer (Amersham Biosciences). Fragment sizes were recorded and analyzed using MegaBACE fragment profiler software version 1.2 and inspected manually. Allele sizes and peak intensities were recorded. Low intensity bands were assigned missing values. Two markers that were monomorphic (GBM1043 & GBM1036) and one marker that amplified multiple fragments (GBM1326) were excluded from further analysis (Table 2, S1 Fig.). Six accessions were excluded from diversity and population differentiation analysis due to pure DNA quality leaving 1485 accessions for analysis (S1 Table).

**Table 2:** Diversity statistics for 1485 landrace accessions.

Marker	Mplex	Dye	SSR	Linkage group	Position	Fragment range	Allele number	Missing (%)	Hetero-zygosity	PIC	MAF	Allelic richness	Gene diversity
			motif										
GBM1363	M1	HEX	(AGG)7	5H	120.68	110–120	3	1.5488	0.0068	0.3776	0.5204	2.1950	0.5014
GBM1404	M1	FAM	TATG	6H	129.76	220–290	8	1.8182	0.0062	0.0499	0.9744	2.7470	0.0506
GBM1461	M1	TAMRA	(CA)6n	1H	135.94	190–240	20	8.0808	0.0022	0.8108	0.2648	12.4300	0.8307
GBM1033	M2	FAM	(AT)9	7H	67.13	270–290	8	1.9529	0.0000	0.5832	0.4849	6.4520	0.6432
GBM1110	M2	HEX	(AAG)6	3H	60.27	210–245	10	1.0101	0.0394	0.5051	0.6360	5.7520	0.5460
GBM1326	M2	TAMRA	(CTT)8	7H	31.24	Excluded							
GBM1013	M3	FAM	(CTG)9	1H	67.5	160–175	5	0.8754	0.0075	0.3542	0.7137	3.7320	0.4231
GBM1015	M3	HEX	ACAT	4H	115.94	190–275	22	1.2121	0.0204	0.8215	0.3361	15.1820	0.8350
GBM1176	M3	TAMRA	AT	5H	18.59	280–295	7	4.0404	0.0000	0.6430	0.3541	5.5940	0.7001
GBM1031	M4	HEX	AG	3H	50.26	280–295	6	0.6061	0.0014	0.6090	0.4383	5.0920	0.6697
GBM1043	M4	FAM	AAC	3H	90.39	Excluded							
GBM1212	M4	TAMRA	(AGG)5	6H	55.1	100–111	5	0.6061	0.0014	0.4857	0.5132	3.3620	0.5740
GBM1003	M5	TAMRA	CTT	4H	79.53	185–220	11	1.4141	0.0075	0.5450	0.5686	6.1160	0.5975
GBM1035	M5	HEX	CT	2H	29.46	270–285	5	1.1448	0.0020	0.7075	0.3188	4.8420	0.7515
GBM1064	M5	FAM	AGGG	5H	157.6	280–300	8	1.0774	0.0000	0.4078	0.7102	4.4570	0.4515
GBM1020	M6	TAMRA	AC	4H	64.05	240–250	4	2.0875	0.0007	0.3811	0.5220	2.4050	0.5033
GBM1036	M6	HEX	CT	2H	156.39	Excluded							
GBM1334	M6	FAM	(GGC)8	1H	70.69	120–140	5	2.3569	0.0014	0.3162	0.7517	2.8890	0.3799
GBM1029	M7	TAMRA	AG	1H	60.42	220–230	5	0.2020	0.0007	0.3768	0.6144	3.0270	0.4825
GBM1047	M7	HEX	AGC	2H	129.66	205–222	6	0.1347	0.0027	0.5792	0.4110	4.3710	0.6494
GBM1413	M7	FAM	(TCATA)6	3H	49.69	150–175	6	0.2020	0.0303	0.5738	0.4674	4.5740	0.6423
GBM1021	M8	FAM	AC	6H	40.17	250–280	15	1.0774	0.0129	0.7313	0.3067	7.9930	0.7677
GBM1060	M8	HEX	GGT	7H	8.78	200–220	6	1.0101	0.0007	0.4343	0.6824	4.5540	0.4832
GBM1075	M8	TAMRA	GT	6H	50.08	290–305	5	0.8081	0.0007	0.4877	0.6330	4.1220	0.5384
GBM1007	M9	FAM	AC	1H	26.45	180–230	22	0.9428	0.0292	0.6932	0.4657	12.8360	0.7212
GBM1256	M9	TAMRA	(GA)8	6H	75.4	340–360	9	1.6162	0.0034	0.8396	0.2128	8.6300	0.8562
GBM1483	M9	HEX	(GCG)7	5H	80.64	150–180	6	1.6162	0.0150	0.3901	0.6253	3.6110	0.4856
GBM1221	M10	HEX	(AC)10	4H	14.65	105–135	12	0.6734	0.0027	0.7304	0.3601	8.9570	0.7628
GBM1405	M10	TAMRA	(CGCA)5	3H	86.33	270–290	4	0.5387	0.0047	0.6853	0.3542	4.0000	0.7335
GBM1516	M10	FAM	CT	7H	81.21	90–115	10	0.5387	0.0419	0.6599	0.3982	7.7430	0.7074
GBM1063	M11	FAM	(ACAT)7	6H	63.49	195–220	7	0.2020	0.0337	0.6864	0.3640	5.6550	0.7326
GBM1280	M11	HEX	CTT	3H	3.83	270–295	6	0.2020	0.0054	0.5970	0.4325	4.1150	0.6639
GBM1323	M11	TAMRA	(GCC)8	4H	28.96	110–135	7	0.4714	0.0020	0.5159	0.5622	4.6990	0.5823
GBM1002	M12	TAMRA	CCT	1H	101.5	250–355	12	0.4714	0.0041	0.3355	0.7727	4.4550	0.3723
GBM1464	M12	FAM	AT	7H	53.53	130–220	15	0.6734	0.0102	0.7254	0.3045	6.7780	0.7641
GBM1501	M12	HEX	(TAGA)6	4H	0	250–290	12	0.6061	0.0264	0.4830	0.6386	4.9560	0.5329
GBM1026	M13	FAM	AC	5H	53.08	210–220	5	2.2222	0.0014	0.3946	0.6687	4.0260	0.4694
GBM1419	M13	TAMRA	CTCAT	7H	95.75	90–130	8	1.6835	0.0164	0.5025	0.6234	5.4250	0.5509
GBM1459	M13	HEX	(AC)7	2H	64.35	150–175	10	2.2896	0.0489	0.6670	0.4542	7.9090	0.7067
GBM1018	M14	FAM	(CCG)6	4H	132.69	250–285	7	1.8182	0.0034	0.3947	0.7023	3.8360	0.4505
GBM1061	M14	HEX	(GGT)6	1H	130.75	320–350	10	5.9933	0.0508	0.6318	0.3865	5.8000	0.6886
GBM1208	M14	TAMRA	(AG)6	2H	102.85	130–160	10	2.2222	0.0034	0.5373	0.5048	6.4780	0.6089
GBM1008	M15	HEX	(AAC)10	6H	95.37	150–180	10	1.6835	0.0397	0.6468	0.3884	7.9290	0.6967
GBM1054	M15	FAM	CCG	5H	132.16	255–275	9	1.8855	0.0034	0.5866	0.5000	5.0010	0.6448
GBM1218	M15	HEX	GA	2H	72.45	130–150	11	2.5589	0.0069	0.5498	0.5729	6.4030	0.5989
Mean							8.8571	1.5280	0.0119	0.5484	0.5122	5.7412	0.6036

Marker name, multiplex (Mplex) number, SSR motif, chromosomal linkage group, genetic position in cM, range of fragment sizes, number of alleles per locus, percentage of missing data per marker, heterozygosity, polymorphic information content (PIC), major allele frequency (MAF), allelic richness and gene diversity values across 45 SSR loci.

### Inferring the population structure

The population structure of the 1485 landraces (Lrc1485) considered was inferred using Structure 2.2 [47], [48] based on 42 SSR markers. This approach uses a Bayesian clustering analysis to assign individuals to clusters (*K*) without prior knowledge of their population affinities. Structure simulations were performed with the number of presumed clusters from *K* = 1 to *K* = 20 and five runs per *K* value. For each run, the initial burn-in period was set to 50,000 followed by 100,000 Markov Chain Monte Carlo (MCMC) iterations. The most probable number of clusters was determined by plotting the estimated likelihood values [LnP(D)] as a function of *K* [48]. Furthermore, delta (*K*) values were also calculated as proposed by [49]. A cut-off limit of 60% membership coefficient (Q-matrix) was considered to assign the individuals to a particular group as suggested by [50]. Accessions that did not meet this criterion were considered as admixed.

Principal Component Analyses (PCA) were performed using Past 2.12 [51]. Neighbor-Joining tree and Neighbor-Net planar graph based on Hamming distances (uncorrected *p*-distance) between 1485 landraces were constructed using SplitsTree 4.13.1 [52].

### Genetic diversity and population differentiation

The number and frequency of alleles, gene diversity and heterozygosity (H_e_), were determined for all loci across the total population using Powermarker 3.25 [53]. Polymorphism Information Content (PIC) values were determined according to [54]. Genetic variation within and among populations was estimated by Analysis of Molecular Variance (AMOVA) using Arlequin 3.1 [55]. AMOVA was conducted between morphological, geographical and Structure inferred groups. Genetic differentiation among groups was calculated based on unbiased F_st_ estimators [56]. Pair wise population comparisons using Fixation statistics (F_st_) were determined among all groups as well as allelic richness, gene diversity (GD) and the number of alleles for each group were calculated using Fstat 2.9.3 [57]. Allelic richness values for subpopulations were calculated based on rarefaction to account for varying sample size [58]. In all analyses, statistical significance was determined by performing 1000 permutations.

### Spatial distribution of groups in relation to geography and climate

Geographic ground distances in kilometres between accessions were calculated based on latitude and longitude coordinates. The genetic diversity index [59] was calculated, and the genetic distance matrix was calculated using the shared allele distance approach of [60] based on allele frequencies at 42 loci. Based on this, Mantel tests were conducted between genetic distance and all other distance matrices. Mantel correlograms were generated analogous to the autocorrelation function [61] - and allowed to assess the overall relationship between matrices and to determine the significance level of correlation for each distance class. Mantel correlograms were constructed using Passage 2.1 [62].

The spatial distribution of accessions, categorized into population-based groups, was visualized in relation to climate zones based on the Köppen-Geiger classification [63]. These climate zones are obtained by classifying the mean climate conditions on land areas around the globe using climate variables such as annual and seasonal mean temperatures and precipitation. The five main zones range from tropical climate through arid and temperate climates to polar climates. Subgroups can be determined by, for example, hot summers, dry and cold winters, year-round precipitation or monsoonal conditions.

### Establishing a Core Reference Set (CRS) for high resolution LD-mapping

To build a legacy CRS [64] comprising c. 600–700 genotypes, we first genetically purified the entire collection using two rounds of single seed descent (SSD) [65] under greenhouse conditions in 2008–2009 (started with the same plants from which we isolated DNA, see above). Afterwards, the materials were multiplied and survey-phenotyped under field conditions at IPK using local standard agricultural practices in two subsequent years: i) 2010 (single row per landrace genotype, isolated by one row of a spring wheat genotype, 8 plants per row, 20 cm distance between the plants), and ii) in 2011 (micro plots, 1.2×1 m; all available seeds from the 2010 harvest were sown and equally distributed over six rows). Plants were harvested and threshed manually to avoid mixtures of seeds. In total, 1014 genotypes produced sufficient seeds after two rounds SSD and the two subsequent multiplication cycles (S1 Table). Subsequently, the M strategy as implemented in Mstrat [66] was used to establish the legacy CRS. All data sets available were considered for this: i) geographical origin; ii) 42 SSR markers, and iii) morphological traits as quantitative parameters, which were obtained during seed multiplication in 2011, such as row type, caryopsis type, heading date, plant height and spike length (S1 Table). The best CRS that maximizes the number of observed alleles was then established based on five replications using Mstrat. Diversity scores calculated based on allelic richness [67] were compared and validated with a random sampling approach as well as by comparing phenotypic diversity.

## Results

We considered a collection of 1485 landraces originating from 41 countries (Fig. 1). This collection comprised of 708 two-rowed (47.7%) and 777 six-rowed (52.3%) barley genotypes. In total, 284 (19.1%) naked genotypes were considered (Table 1).

### High levels of polymorphism and many rare alleles in the collection

Data quality was high, and the level of missing information across SSR markers was very low (1.528%). Based on the 42 SSR markers considered, 372 alleles were obtained with fragment sizes ranging from 90 to 360 bp (Table 2, S2 Table). The number of alleles per locus ranged from 3 (GBM1363) to 22 (GBM1007, GBM1015) with an average of 8.86 alleles per locus. Major allele frequencies per locus ranged between 0.213 (GBM1256) and 0.974 (GBM1404) (mean value of 0.512) and PIC values ranged from 0.050 (GBM1404) to 0.839 (GBM1256) (average of 0.548). The majority of markers (78%) showed PIC values between 0.4 and 0.8. The average allelic richness was 5.7412, ranging from 2.1950 (GBM1363) to 15.1820 (GBM1015) (Table 2). An average gene diversity (GD) value of 0.6036 was obtained, indicating a high level of genetic variation among the accessions. Heterozygosity levels (He) were very low ranging 0–0.05, with an average value of 0.0119 per locus (Table 2). A total of 157 rare alleles (allelic frequency <1% in the total collection) were identified amounting to 42% of the total number of alleles discovered (Table 2, S2 Table).

### Population structure within the panel

**Figure 2:**
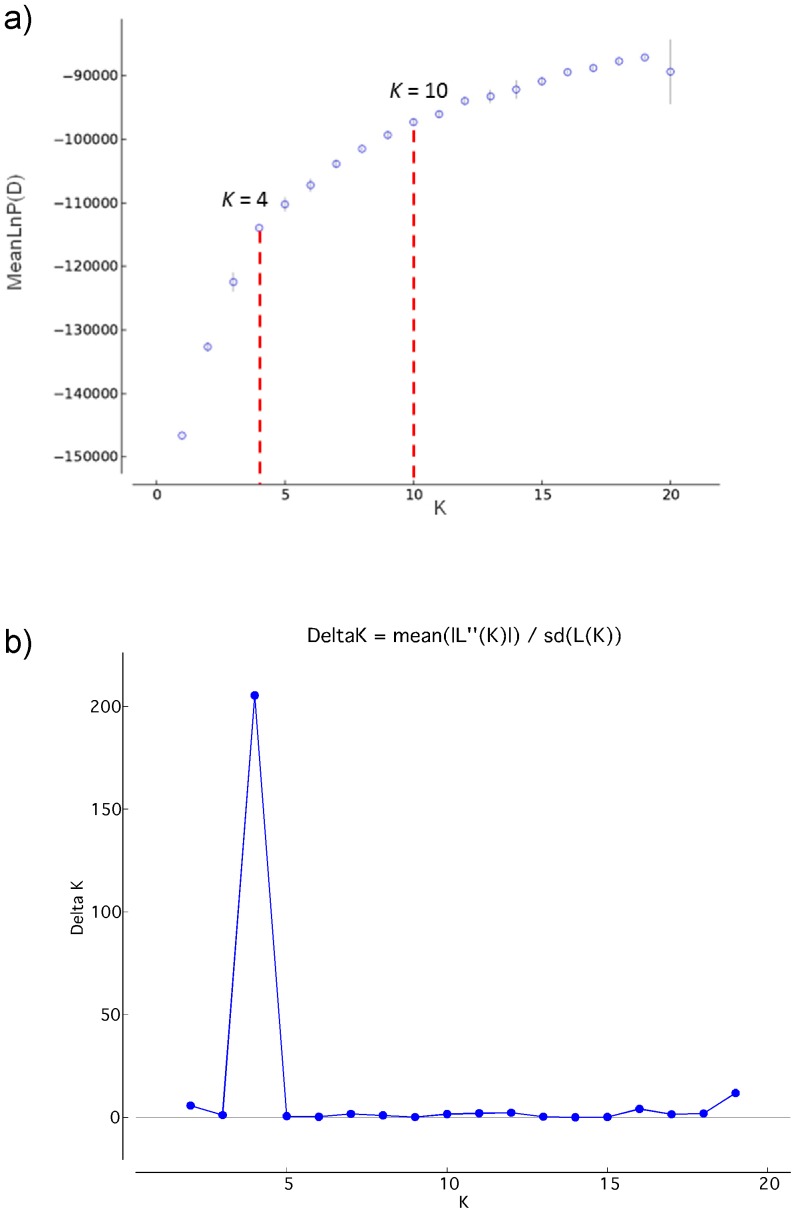
Structure analysis for 1485 barley landraces. **a)** mean Log probability values (LnP(D)) plotted as function of *K* (number of clusters); **b)** Delta *K* vs. *K* plotted as proposed by [44]. The graph indicates the maximum change at *K* = 4.

Structure runs were performed for *K* = 1 to *K* = 20 based on the distribution of 372 alleles at 42 SSR loci among 1485 accessions. LnP(D) values increased slowly starting from *K* = 10, thus probably representing the most appropriate number of major clusters in this collection. However, the maximum delta (*K*) value was reached at *K* = 4 (Fig. 2).

The primary division at *K* = 2 was observed mainly between Ethiopian (*Group 1*) and non-Ethiopian landraces (*Group 2*) (S1 Table). Here 92.59% of the landraces were assigned to either of two groups (*G*) considering the 60% membership coefficient (Q-matrix). The least number of admixtures (110 accessions; 7.4%) was observed at this *K* value.

**Figure 3:**
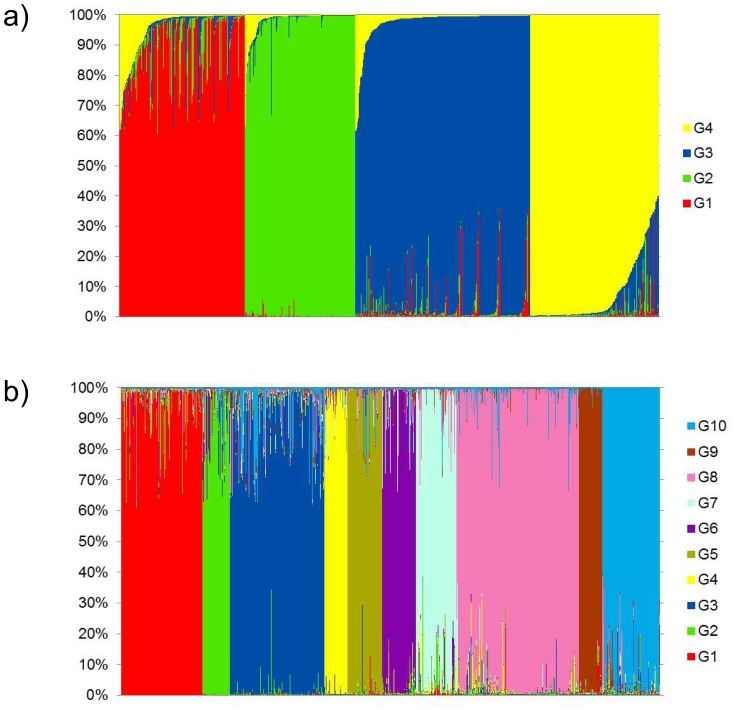
Population structure of 1485 landraces using 42 SSR inferred by Structure. **a)** for *K* = 4; and **b)** for *K* = 10. Genotypes were ordered according to their membership coefficient (Q) values to one group.

At *K* = 4, 91.1% of the landraces were assigned to one of the following groups – *G1* (314): two-rowed and six-rowed barleys mainly from Southwest Asia; *G2* (277): two-rowed and six-rowed landraces mostly from Ethiopia; *G3* (439): mainly six-rowed hulled barleys from a wide geographical range including northern Africa; and *G4* (323): mainly two-rowed hulled forms from Europe and Southwest Asia. The population structure inferred using PCA provided congruent results (Fig. 1, Fig. 3, S2 Fig., S3 Fig., Table 3, Table 4, S1 Table).

**Table 3:** Assignment of 1485 landraces into Structure inferred groups.

		Accessions	Hulled		Naked	
	Group	assigned	Two-rowed	Six-rowed	Two-rowed	Six-rowed
a)	G1	314	108 (34.30)	151 (48.00)	43 (13.60)	12 (3.80)
	G2	277	44 (15.80)	36 (12.90)	89 (32.10)	108 (38.90)
	G3	439	13 (2.90)	420 (95.60)	1 (0.20)	5 (1.10)
	G4	323	316 (97.80)	3 (0.00)	3 (0.00)	1 (0.00)
	Admixed	132	84 (63.60)	24 (18.10)	7 (5.30)	17 (12.80)
	Total	1485	565 (0.380)	634 (0.426)	143 (0.096)	143 (0.096)
b)	G1	194	0 (0.00)	0 (0.00)	88 (45.30)	106 (54.6)
	G2	66	1 (15.00)	64 (96.90)	0 (0.00)	1 (1.50)
	G3	226	8 (3.50)	218 (96.40)	0 (0.00)	0 (0.00)
	G4	56	33 (58.90)	23 (41.00)	0 (0.00)	0 (0.00)
	G5	83	43 (51.80)	40 (48.10)	0 (0.00)	0 (0.00)
	G6	80	1 (1.20)	70 (87.50)	0 (0.00)	9 (11.20)
	G7	97	91 (93.80)	6 (6.10)	0 (0.00)	0 (0.00)
	G8	295	288 (97.60)	3 (1.00)	4 (1.30)	0 (0.00)
	G9	54	0 (0.00)	0 (0.00)	42 (77.70)	12 (22.20)
	G10	138	5 (3.60)	132 (95.60)	0 (0.00)	1 (0.72)
	Admixed	196	95 (48.40)	78 (39.70)	9 (4.50)	14 (0.71)
	Total	1485	565 (38.00)	634 (42.60)	143 (9.60)	143 (9.60)

(a) groups at *K* = 4 and (b) at *K* = 10. Assigned - accessions with membership coefficient above the threshold of 60%. Admixed - accessions with less than 60% membership coefficient to a particular group. Numbers in brackets represent the proportion of accessions assigned to either group in percentage (%).

**Table 4:** Diversity and summary statistics for Structure inferred groups a) *K* = 4; and b) *K* = 10.

	Group	Sample size	Major allele frequency	Allele No.	Mean allele No.	Availability	Gene diversity	Hetero-zygosity	Allelic richness	Group specific rare alleles
**a)**	G1	314	0.5553	**296**	7.0476	0.9857	0.5750	0.0097	6.6743	45
	G2	277	0.7506	**206**	4.9048	0.9862	0.3301	0.0123	6.5072	57
	G3	439	0.5744	**296**	7.0476	0.9794	0.5439	0.0147	7.2848	77
	G4	323	0.7029	230	5.4762	0.9882	0.4084	0.0082	6.2770	57
**b)**	G1	194	0.7912	174	4.1429	0.9872	0.2805	0.0121	3.242	57
	G2	66	0.6251	189	4.5000	0.9838	0.4816	0.0164	4.439	5
	G3	226	0.6460	**252**	6.0000	0.9802	0.4674	0.0149	4.917	**78**
	G4	56	0.7759	140	3.3333	0.9868	0.3006	0.0052	3.323	5
	G5	83	0.8150	146	3.4762	0.9845	**0.2589**	0.0122	3.309	5
	G6	80	0.6315	204	4.8571	0.9836	0.4810	0.0097	4.684	6
	G7	97	0.6127	213	5.0714	0.9880	**0.4929**	0.0114	4.774	9
	G8	295	0.7126	218	5.1905	0.9884	0.3947	0.0084	3.946	70
	G9	54	0.7218	**129**	3.0714	0.9868	0.3606	0.0080	3.065	8
	G10	138	0.6649	220	5.2381	0.9781	0.4428	0.0114	4.663	48

*alleles <1% frequency in the whole collection are considered rare alleles.

Known key determinants of the population structure for barley like i) row type of the ear, ii) kernel coverage, and iii) geographical origin divided the collection at *K* = 10 (Fig. 1, Fig. 3, S2 Fig., Table 3, Table 4, S1 Table). The clusters were also relatively well associated to distinct climate zones (S4 Fig.). *G1* (194) consisted of naked barleys mostly from Ethiopia. *G2* comprised of 66, mostly six-rowed landraces from a wide geographical range were hot desert climate is prevailing. *G3* was the second largest group (226; 15.2%) and harboured the highest average number of alleles (252) and 78 group specific rare alleles. Allelic richness based on rarefaction provided the highest value for this group (Table 4b). Mainly six-rowed hulled barleys from the Mediterranean were included here: Libya (61), Morocco (48), Spain (30), Italy (21), Greece (28) and Turkey (10). All 56 landraces assigned to *G4* originated from or close to Georgia. Interestingly, both two-rowed and six-rowed hulled types were present in this group. All but one of the 83 accessions assigned to *G5* were hulled barleys from Ethiopia but with different row types: including all assigned *deficiens* (9); *intermedium* (4); and *labile* (7) genotypes. Gene diversity was lowest for *G5* (0.258) (Table 4). Altogether, 80 landraces clustered in *G6*, comprising mainly hulled barley from Afghanistan (61) and Iran (7). Among nine naked barleys considered here, five were collected in Afghanistan. Ninety-seven, mainly two-rowed landraces from Minor Asia and the eastern part of the Fertile Crescent were assigned to *G7* including lines from Turkey (29), Iraq (11), Iran (32), Afghanistan (5) and Georgia (7). Highest gene diversity value was found for this group (0.492) (Table 4). *G8*, the largest group at *K* = 10, consisted of 295 accessions of mainly two-rowed hulled types. Among them, 276 accessions originated from Europe. *G9* was the smallest group harbouring 54 naked landraces, mainly of non-Ethiopian origin (52), and only 129 alleles (Table 4). Finally, *G10* consisted of 138 landraces, majorly six-rowed hulled barleys from a broad geographical range north of the Mediterranean Sea (Fig. 1, S4 Fig.). At *K* = 10, principal components (PC) 1 and 2 explained 9.90% and 8.58% of the variation, respectively, and separated most Structure inferred groups (S2 Fig.). Relationships among accessions within each Structure inferred group are shown in S5 Fig. Variation explained by PC1 and PC2 together, per group, ranged from 11% to 51% (*G1*, 23.95%; *G2*, 39.67%; *G3*, 11.17%; *G4*, 25.32%; *G5*, 21.15%; *G6*, 18.036%; *G7*, 26.04%; *G8*, 19.99%; *G9*, 51.05%; *G10*, 16.84%) indicating especially within *G1*, *G2*, *G4*, *G5*; *G7* and *G9* further substructure based on e.g. row type or geographical origin.

Pairwise F_st_ values among Structure inferred groups at *K* = 10 ranged from 0.196 (between *G4* - from Georgia and *G7*) to 0.556 (between *G1* – naked Ethiopian, and *G4*). Similarly pairwise F_st_ comparisons for inferred groups at *K* = 4 ranged from 0.187 to 0.388 (S3 Table). Highly significant levels of genetic differentiation between and within populations were found using AMOVA. The percentage of genetic variation among populations ranged from 6.38% (all two-rowed vs. all six-rowed Ethiopian) to 37.6% (Structure inferred groups^(*K = 10*)^) and the percentage within the populations ranged from 62.40% (Structure inferred groups^(*K = 10*)^) to 93.62% (all two-rowed vs. all six-rowed Ethiopian). The values for the comparison of *all Ethiopian naked vs. all non-Ethiopian naked* barleys are interesting and possibly indicating two evolutionary lineages (S4 Table).

### Association between eco-geographical factors and genetic diversity

Significant relationships were found between genetic distances of accessions and eco-geographical parameters at the site of origin (S5 Table). Most significantly correlated with genetic distances was geographical distance, followed by latitudinal distance (S5 Table). The Mantel test between the genetic distance matrix (shared allele distance matrix) and the geographical distance matrix revealed a significant Mantel correlation of 0.357 (*P* <0.0001). The correlation between genetic distance and longitudinal distance (r = 0.305, *P* <0.0001) was high. The correlation between genetic distance and latitudinal differences (r = 0.193, *P* <0.0001) was 2-fold lower than the correlation between genetic and geographic distances. In the Mantel correlogram for genetic distance *vs.* geographic distance, the matrix was subdivided into 20 discrete distance classes. Within the distance class of 0-300 km between the collection sites of accessions, the correlation was highest (r = 0.523). The r - values (Mantel correlation) and their significances declined with increasing distances. Spatial Mantel correlograms provided similar trends (S6 Fig., S5 Table).

Structure inferred clusters at *K* = 10 were assigned to distinct Köppen climates, although value should not be given to the few suspect lines (see below) (Fig. 1, S3 Fig., S4 Fig.). S6 Table shows the major Köppen climates for each of the Structure inferred group. Climate zones range from winter dry tropical climates (Aw) to fully humid boreal climates (Dfb). Most samples are located in warm temperate climates, either summer dry (Csa) or fully humid (Cfb). In more detail one can observe, that all accessions from *G1* and *G5* are located in winter dry climates in Ethiopia; *G4* lines were only sampled from Georgia, where fully humid boreal climates (Dfb) prevail. *G2* is mainly located in hot and dry desert climates (BWh) and *G6* (six-rowed) prevails in wintercold steppe climates (BSk). *G7* occurs along a narrow latitudinal range (30–43° N) in Minor Asia and the Fertile Crescent in warm temperate summer dry climates (Csa). *G8* (two-rowed) and *G10* (six-rowed) were sampled further north, majorly between 40–50°N (Cfb, temperate fully humid) and 36–47°N (Csa, temperate summer dry), respectively.

### Establishment of a core reference set

To determine the theoretical optimum size of a core group representing most of the genetic diversity within the whole Lrc1485 collection, different sizes of core groups from n = 1 to n = 1485, with a step size increase of 50 accessions were computed using Mstrat and plotted against their diversity score calculated based on allelic richness. The M strategy performed better in efficiently capturing maximum allelic diversity than the random sampling approach (S7 Fig., Table 5). The theoretical optimum size of the core group was determined as in the range between c. 600 to 750 samples. More specifically, 745 individuals must be selected to harbour the maximum allelic diversity (S7 Fig.). Subsequently, based on the 1014 genotypes, which were available after two rounds of SSD and multiplication, the core reference set Lrc648 was established using Mstrat consisting of 308 two-rowed (242 hulled and 66 naked types) and 340 six-rowed (285 hulled and 55 naked types) genotypes (S8 Fig., S1 Table, S7 Table). We validated the superiority of the M strategy by comparing phenotypic diversity between the core set Lrc648 and a random set of landraces comprising the same number of accessions (Lrc648r) (S8 Table).

**Table 5:** Comparison of diversity statistics for different panels.

	Genobar collection	Lrc1485	Lrc648	Lrc648r	Lrc648 2-rowed	Lrc648 6-rowed
Number of accessions	224	1485	648	648	304	344
Number of SSR markers	42	42	42	42	42	42
Average allele number	5.36	**8.95**	8.38	7.54	7.52	7.00
Gene diversity	0.57	0.60	0.60	0.59	0.61	0.58
Polymorphism information content	0.52	0.55	0.55	0.54	0.56	0.52
Total number of alleles	225	**372**	352	317	316	294
Major allele frequency	0.57	0.51	0.51	0.51	0.51	0.53
Number of unique alleles	53^a^	159^a^	141^a^	135^ a^	112	97
Number of rare alleles (freq <0.01)	31	**157**	138	105	90	95

Genobar, Lrc1485, Lrc648, Lrc648r (648 randomly selected lines of Lrc1485 as additional validation), Lrc648 two-rowed sub-panel, and Lrc648 six-rowed sub-panel. ^a^two way comparisons: Genobar *vs* Lrc1485, Genobar *vs* Lrc648, Genobar *vs* Lrc648 (304 two-rowed accessions) and Genobar *vs* Lrc648 (344 six-rowed accessions) and Genobar *vs* Lrc648r.

## Discussion

The demand for higher yielding and better-adapted crop varieties has raised the need to exploit the large *ex situ* genebank collections [68]. So far, in case of barley, only very few collections of landraces were investigated, mostly sampled from particular geographical regions [34], [35], [69], [70]. The primary aim of this study was to establish and to characterize a diverse legacy collection of barley landraces adapted to a wide range of climates.

### Lessons learned from genebank materials

Most probably due to careful selection and accurate description of the material (conservation management of IPK genebank certified according to - DIN EN ISO 9001:2008; line splitting practice for heterogeneous accessions), very few geographical outliers were observed. Another reason for the good fit of accessions into geographical clusters could be that 1035 (69.7%) accessions were sampled during IPK collection missions (or from collections which are hosted at IPK e.g. AUTMAYR-22-32, HIND-35/36, IRNFAOKU-52-54, S1 Table) and then maintained at IPK – thus significantly reducing confusions arising from seed exchange with other genebanks. For most accessions considered here, the collection site information was available (which is usually rarely the case for landraces from other genebanks), thus significantly improving spatial-evolutionary studies, too.

Few accessions appeared to be suspect and do not seem to represent their original collection site (e.g. at *K* = 10 for *G1*: the seven lines sampled in Europe and Morocco). These accessions were probably not collected originally in these areas. We assume they were sampled elsewhere and then grown *ex situ* in these countries, or they may have been incorrectly recorded or mixed up during seed exchange and propagation. As recently shown by [50] consequent elimination of any doubtful line identified would provide the best resolution.

### Higher values of genetic diversity within the Lrc1485 and the legacy collection Lrc648 compared to the world-wide Genobar GWAS panel

In the collection of 1485 barley landraces, 372 alleles were detected using 42 SSR markers, with an observed average allele number (AN) of 8.95 alleles per locus, which is larger than in most other studies, including [71] using 45 SSRs in 223 cultivars of worldwide origin but also including some wild barleys (AN = 7.7), [69] using 39 SSRs for Eritrean landraces (AN = 7.6), [35] with 44 SSRs for Himalayan landraces (AN = 5.54) or [72] with 12 SSRs for Sardinian landrace populations (AN = 5.6). On the other hand, [73] reported AN = 16.7 for a worldwide collection of 953 barley accessions including wild barleys based on 48 genomic SSRs – which are much more polymorphic than the cDNA derived SSRs in our study. Certainly, the number of alleles per locus depends on the genotypes considered, the loci investigated and the marker type.

A total of 157 rare alleles were detected in the whole collection. Rare alleles were mostly detected at SSR loci, which displayed a high number of polymorphic alleles. The presence of 42% rare alleles highlights the potential of this collection for subsequent allele mining studies but could potentially limit GWAS.

To evaluate the potential of Lrc1485 for GWAS in more detail, genetic diversity values were directly compared to the potentially most diverse GWAS panel for barley comprising of 224 spring barley cultivars and landraces of worldwide origin (“Genobar” panel) [32], [46], [74], [75] - using the same set of 42 SSR markers (Table 5). Overall, the Lrc1485, the CRS Lrc648 but also the subpanels of Lrc648 based on the row type of the ear - are all more diverse than the Genobar panel as indicated by e.g. the total number of alleles, number of unique alleles or average gene diversity. Unique alleles observed in the Genobar panel came from genotypes collected from East Asia as well as from the Americas. Such regions were not considered in Lrc1485 (Table 5).

LD estimates between SSR markers were computed (S9 Fig.). All pair-wise comparisons showed very low LD (r^2^ <30), which is not surprising due to the relatively low marker coverage (S1 Fig.) and the large population size. Inferring genome wide LD dynamics in this population from few SSR markers would be a vague interpretation. Therefore, high-density marker coverage across the genome is required (a least a few hundred of mapped and equally spaced SNPs) to estimate more precisely the LD extent and the pattern at the population level and across the genome.

### Ten major clusters define most of the population structure within the spring Lrc1485 panel

We assessed population structure by different approaches. Based on Bayesian clustering and PCA analyses we considered *K* = 10 as the most appropriate number of major clusters in the Lrc1485 collection. Although the maximum delta (*K*) value was reached at *K* = 4, clusters at *K* = 10 were better defined based on geographical origin, row type of the ear and caryopsis type – and also associated to distinct Köppen climate zones.

Clusters *G1*, *G3*, *G4*, *G5 G8* and G10 were distinct and well supported. An intrinsic genetic substructure was visible for groups, which included accessions from a larger geographical range (*G2*, *G6*, G7, *G9*). PCA provided mostly congruent results for these clusters. However, PCA does not classify accessions into discrete clusters in all cases, especially not when admixed accessions and accessions of various geographical origins with a constant gene flow are included [76]. Neighbor-Joining and Neighbor-Net analysis supported these findings. All clusters obtained by Structure analysis were distinguishable, although a high amount of reticulation was evident, owing to the fact that common alleles per locus were shared among geographic regions (S10 Fig., S11 Fig.).

To explore the genetic diversity and relationships among and within Structure inferred groups, various diversity statistics were assessed (Table 4). Gene diversity (GD) over 42 loci was highest for *G7*, *G6*, *G2* and *G3*, which is comparable with other local collections [69], [77]. Highest values for allelic richness and group specific rare alleles were found in *G3*, which might be due to i) materials sampled from a wide range of eco-geographical conditions around the Mediterranean Basin (local climates and niches at similar latitudes) (Fig. 1, S4 Fig.); or ii) gene flow among barley genepools [31].

As expected for barley, the row type of the ear was an important determinant of the population structure [28], [78] and six groups comprised majorly either two-rowed (*G7*, *G8*) or six-rowed (*G2*, *G3*, *G6*, *G10*) types, respectively (Table 3). Karyopsis type defined *G1* and *G9* as they included only naked types. Adaptation to local climates is defined e.g. through *G4* (from Georgia) and *G5* (from Ethiopia) containing both row types. However, within groups *G1*, *G4*, *G5* and *G9* subclusters can largely be explained by the row type of the ear.

### Eco-geographical factors and spatial genetics

Detailed knowledge of environmental parameters at a certain collection site can help determining the role of relevant climatic factors influencing the genetic differentiation and adaptation of genotypes to their environments. Furthermore, this knowledge might help selecting most suitable parental lines for population development and breeding programs.

The distribution of accessions was found rather along the latitudes, meaning a wider W-E than N-S window. Just Ethiopia is the southern-most sampling area, i.e. stretching the latitudinal direction the most. In general, climatologically, climate zones are determined by i) incoming solar radiation (more at equator, less at poles); ii) b) altitude (higher elevation will yield similar climate zones as usually on higher latitudes); and iii) proximity to the sea yielding maritime *vs*. continental climates. Thus the general pattern of climate zones (as the word “zonal” means in this context) is band along the latitudes.

The distribution pattern of Structure inferred groups probably indicate a preferred distribution path rather zonal (W-E) than meridional (N-S), i.e. higher correlation with longitude and indeed this was observed for Lrc1485 groups. As domesticated barley spread meridionally from the Fertile Crescent to north-western Europe, the crop encountered considerable ecological and environmental change. Natural mutation, selection and enrichment of favorable alleles at key loci such as *Ppd-H1* [79], *HvCEN* [80] or vernalization-related genes *VRN-H1* [81], [82], *VRN-H2* [83] and *VRN-H3* (also known as *HvFT1*, [84]) contributed to successful environmental adaptation and range extension in barley.

However, based on the IPK genebank information system, we selected only spring barleys, which flowered at IPK without the need for any cold period to promote flowering. In Central Europe (such as at IPK), spring types are sown between early March and end of April depending on the weather conditions every year [68]. Thus, vernalization-or frost tolerance-related loci should be less relevant for the adaptation of spring barleys to their environments. However, as shown by [85], the Genobar spring barley collection, harbored 8 haplotypes at *VRN-H3*. Genotyping the same set of 224 accessions at *VRN-H2* suggested the presence of recessive alleles at *VRN-H2* (Kilian et al. unpublished), observed as deletions of a cluster of up to three ZCCT family genes, which contribute to the spring growth habit [86]. Thus, ideally, genotypes should be characterized at molecular and phenotypic levels, before assigning them to a specific growth habit (i.e. winter, facultative, spring). However, in the genebank context this has not been achieved for most of the collections yet. Therefore, we expect some facultative types within Lrc1485.

As already shown partly for the Genobar collection, which harbors six haplotypes at *PpdH1* [85] and six out of seven haplotypes detected in the domesticated genepool at *HvCEN* studied by [80], we also expect a remarkable number of haplotypes at key genes responsible for environmental adaptation within Lrc648. Thus, we suggest using the legacy CRS Lrc648 or their two subpanels in particular for allele mining and gene discovery studies.

Although altitude must be considered as an important factor influencing the genetic diversity distribution [87], this factor was not considered in our studies because precise geographic coordinates were not available for all landraces.

The initial Köppen analysis presented here does not give a very clear picture due to outliers and thus a rather high within-group-variability (S6 Table). However for some groups a particular climate applies. Regarding climate change one could investigate Köppen maps based on future climate projections and check where climate zones suitable for barley cultivation will be located in the future. Modelling crop performance under changing climates will help guiding the breeding programs to the expected future needs [88]–[90].

### New insights into barley evolution: two examples

With our genetic analysis in 1485 barley landraces, new insights into barley domestication history can be obtained. In total, 299 accessions from Ethiopia were genotyped (Table 1), thus providing probably the largest SSR data set generated for Ethiopian landraces so far (S1 Table, S2 Table). Overall, Ethiopian barleys were apparently found to be distinct from all other groups (S3 Table, S4 Table), which is in line with previous studies [87], [91]–[93]. Different evolutionary forces (environment) and domestication histories (e.g. agricultural practices, cultural preferences of human tribes) in the Ethiopian highlands compared to the Fertile Crescent might be reasons for this distinctness [5], [94]. At *K* = 10, 277 Ethiopian landraces were assigned to either of two groups comprising naked (*G1*, 194 lines) and hulled types (*G5*, 83 lines). Both groups were further sub-structured according to their row type of the ear (S5 Fig.).

Although morphologically diverse, Ethiopian hulled barleys harboured a relatively low level of nucleotide diversity as indicated by the lowest gene diversity value, the second lowest level for allelic richness and only five group specific rare alleles. These results provide further evidence that Ethiopian barleys went through a major genetic bottleneck followed by adaptation to climatic (e.g. rainfall patterns, altitude) and edaphic conditions in the Abyssinian highlands (Table 4, S6 Table) [87], [95]–[98]. It is interesting that the early flowering haplotype IV at *HvCEN* (which derived from major haplotype II) predominated in genotypes assigned to *G5* (89%) [80]; S1 Table). Furthermore, based on our preliminary data set at *PpdH1* (Sharma et al. unpublished), most lines from *G5* carry insensitive alleles, thus in combination with haplotype IV at *HvCEN*, providing favorable alleles for the two growing seasons of barley in Ethiopia - *Meher* and *Belg* [87].

Interestingly, Ethiopian naked barleys (*G1*) harbored 57 group specific alleles (second largest number found) and higher value for allelic richness compared to *G9*. Pairwise comparison of F_st_ values between Structure inferred groups showed that *G1* is most closely related to *G5* (0.35) supporting a common origin of Ethiopian barleys. However, *G1* and *G9* (the two naked groups) are genetically less related (0.49), while *G9* is closer connected to *G7* (F_st_ 0.29). Thus, our data suggest at least two evolutionary lineages of naked barleys, both of which probably originated in the eastern Fertile Crescent from a monophyletic natural mutation (17 kb deletion harboring an *ethylene response factor* (ERF) family transcription factor gene) at the *nud* locus on chromosome 7H [99]–[101]. However, resequencing larger germplasm sets at the *nud* locus are required to test this hypothesis and to shed more light on the origin of naked barley. Interestingly, outside Ethiopia, two-rowed naked landraces were mainly sampled from Iraq and Iran, while six-rowed naked types were collected further east (Afghanistan) [102], [103].

Interestingly, the second highest variation explained among groups by AMOVA was found between *all Ethiopian naked vs. all non-Ethiopian naked* barleys (32.02%) (and thus supporting two evolutionary lineages for naked barley), while *all Ethiopian hulled vs. all non-Ethiopian hulled* barleys explained only 18.50% (S4 Table). AMOVA of *all hulled vs. all naked types* explained 16.17% of variation, which is nearly two-fold higher compared to the variation between *all two-rowed vs. all six-rowed* types (8.87%). Also AMOVA of *all hulled Ethiopian vs. all naked Ethiopian* types (26.69) compared to *all two-rowed Ethiopian vs. all six-rowed Ethiopian* landraces (6.38) suggested that hulled and naked genepools are genetically more distant than the two-rowed and six-rowed clusters. Hulled and naked types probably evolved largely independent under cultivation and were domesticated for different end-use qualities [35].

## Conclusions

The Lrc1485 harbors great genetic diversity. However, the collection size of 1485 genotypes is not manageable in most phenotypic studies, and smaller panels are needed. The legacy CRS Lrc648 established here is best suited for multi-environmental field testing under various climates. However, to work with even more manageable sets, we suggest dividing the Lrc648 into two-rowed and six-rowed subpanels, depending on the trait of interest (Table 5). Increased marker coverage [80] and precise phenotypic data for Lrc648 will help to identify candidate genes also for agronomic and adaptation-related traits using GWAS [27]. Re-sequencing candidate genes [104], [105] or genomic regions underlying quantitative traits using next generation sequencing (NGS) approaches [106]–[109] can be applied for Lrc648.

Seeds of the Lrc648 can be requested in small quantities from IPK. Seed delivery just awaits the Standard Material Transfer Agreement (SMTA) procedure.

## Supporting Information

S1 Fig **Distribution of 45 SSR markers used across the seven linkage groups of barley.**
(TIF)

S2 Fig **Scatter plot of 1485 barley landraces based on Principal Component Analysis calculated from 42 SSR data. a)** for *K* = 4, **b)** for *K* = 10. Colours correspond to the different Structure inferred groups.
(TIF)

S3 Fig **Geographical distribution of 1485 landraces over Köppen climate zones according to Structure inferred groups at** ***K*** **= 4.** Each group (*G1*-*G4*) and admixed types were separately plotted. **(a)** *G1*; **(b)** *G2*; **(c)** *G3*; **(d)** *G4*; **(e)** admixed types. Climate abbreviations are explained in S6 Table.
(TIF)

S4 Fig **Geographical distribution of 1485 landraces over various Köppen climate zones according to Structure inferred groups at** ***K*** **= 10.** Each group (*G1*-*G10*) and admixed types were separately plotted. **(a)** *G1*; **(b)** *G2*; **(c)** *G3*; **(d)** *G4*; **(e)** *G5*; **(f)** *G6*; **(g)** *G7*; **(h)** *G8*; **(i)** *G9*; **(j)** *G10*; **(k)** admixed types. Abbreviations are explained in S6 Table.
(TIF)

S5 Fig **Individual PCA's for each Structure inferred group at** ***K*** **= 10**. Each plot represents a single group: **(a)** *G1*, **(b)** *G2*, **(c)** *G3*, **(d)** *G4*, **(e)** *G5*, **(f)** *G6*, **(g)** *G7*, **(h)** *G8*, **(i)** *G9*, **(j)** *G10*. Blue circles indicate two-rowed and red circles six-rowed barleys.
(TIF)

S6 Fig **Correlograms showing spatial genetic autocorrelation patterns among**: **(a)** genetic distance and geographical distance; **(b)** genetic distance and longitude; **(c)** genetic distance and latitude; **(d)** genetic distance and annual mean temperature; **(e)** genetic distance and mean diurnal range; **(f)** genetic distance and mean temperature of warmest quarter; **(g)** genetic distance and annual precipitation. The x-axis represents distinct classes and the y-axis represents the mantel r values.
(TIF)

S7 Fig **Comparing the sampling efficiency based on Mstrat and random sampling to capture most efficiently genetic diversity to establish a core reference set**. Average diversity score calculated based on allelic richness was plotted against the sample size. Red circles indicate scores of the core collection using the M strategy and blue circles indicate scores of randomly selected accessions.
(TIF)

S8 Fig **Scatter plot of Lrc1485 and Lrc648 based on PCA calculated from 42 SSR data.** Landraces selected for Lrc648 are indicated in red colour.
(TIF)

S9 Fig **Linkage Disequilibrium (LD) display of 1485 barley landraces using 42 SSR markers**. LD was calculated in TASSEL 2.1(www.maizegenetics.net/tassel) using 1000 permutations. Markers are arranged according to the genetic positions on barley genome (see S1 Fig.).
(TIF)

S10 Fig **Evolutionary relationships of 1485 barley landraces I.** The evolutionary history was inferred by a) a Neighbor-Joining tree, and b) a Neighbor-Joining strict consensus tree computed in SplitsTree software.
(TIF)

S11 Fig **Evolutionary relationships of 1485 barley landraces II.** The Neighbor-Net planar graph of uncorrected p-distances visualizes the high amount of reticulation in the collection (Taxa = 1485; Chars = 372; Fit = 90,615; Splits = 4604).
(TIF)

S1 Table **The Lrc1485 collection.** **a)** Details of accession names, their taxonomical designations, row type and kernel coverage, collection sites, collection missions and all other information available from IPK genebank documentation System (GEBIS); and **b)** Structure assignments to groups *K* = 2 to *K* = 20, phenotypic data for Lrc648 and haplotype information for *HvCEN* obtained from [74], if available are given. Accessions selected for the CRS Lrc648 are indicated. Six accessions were excluded from further analysis due to large extent of missing values. NS – not selected for core group of 648 genotypes; *lost during single seed descent and multiplication.
(XLSX)

S2 Table **Final SSR data set for the whole collection of 1491 barley landraces investigated.** Fragment sizes and 0/1 matrix are provided.
(XLSX)

S3 Table **Pairwise comparison of F_st_ values between the Structure inferred groups groups a)** for *K* = 4 and **b)** for *K* = 10. Significance of *P*-values computed after 1000 permutations are represented above diagonal and the F_st_ values are presented below.
(DOCX)

S4 Table **Analysis of Molecular Variance (AMOVA).** Summary of partitioning of genetic variation among and within different groups of Lrc1485.
(DOCX)

S5 Table **Mantel correlogram tables.** **a)** Mantel correlogram tables between: genetic distance and geographic distance; **b)** genetic distance and longitude difference matrix; **c)** genetic distance and latitude difference matrix; **d)** genetic distance and annual mean temperature; **e)** genetic distance and mean diurnal range; **f)** genetic distance and temperature of warmest quarter; and **g)** genetic distance and annual precipitation. ^1^different distance classes are shown; ^2^lower and upper boundary values for each class; ^3^number of pairs for which the correlation was calculated within each distance class; ^4^the mantle correlation for each class; ^5^the significance of mantel correlation for each class.
(DOCX)

S6 Table **Overview of Köppen climates prevailing in Structure inferred groups.** Variety - number of different climates within the group; *the few geographical outliers were not removed. (XLSX)
(XLSX)

S7 Table **Comparison of diversity statistics for different sample sizes of core groups generated from 1485 accessions as well as Lrc1485, Lrc1014, Lrc648 and Lrc648r using 42 SSR markers and climatic variables.** N - number of accessions; AN - average allele number; GD - gene diversity; PIC - polymorphism information content; MAF - average major allele frequency.
(DOCX)

S8 Table **Comparison of phenotypic diversity between the core set Lrc648 (based on Mstrat) and the random set Lrc648r.** Min. - minimum; Max. – maximum; SD. - standard deviation of the measured traits heading date (Hd) (in days to flowering), spike length (Sl) (in cm) and plant height (Ht) (cm).
(DOCX)
