# Supplementary figures and images for: Genetic Diversity and Population Structure in a Legacy Collection of Spring Barley Landraces Adapted to a Wide Range of Climates
Source: PLoS One. 2014 Dec 26;9(12):e116164. doi: 10.1371/journal.pone.0116164 (PMC4277474; doi:10.1371/journal.pone.0116164)

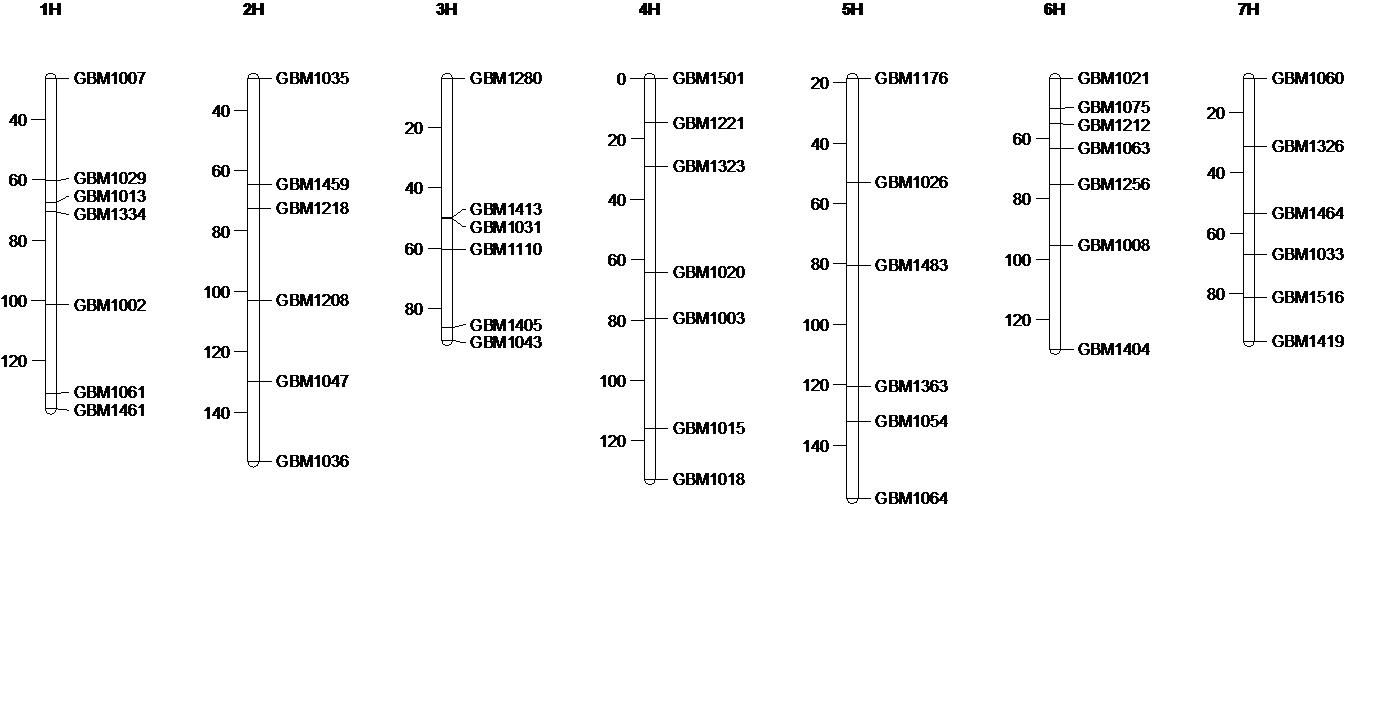

Supplement: S1 Fig — Distribution of 45 SSR markers used across the seven linkage groups of barley. (TIF) [file pone.0116164.s001.tif]

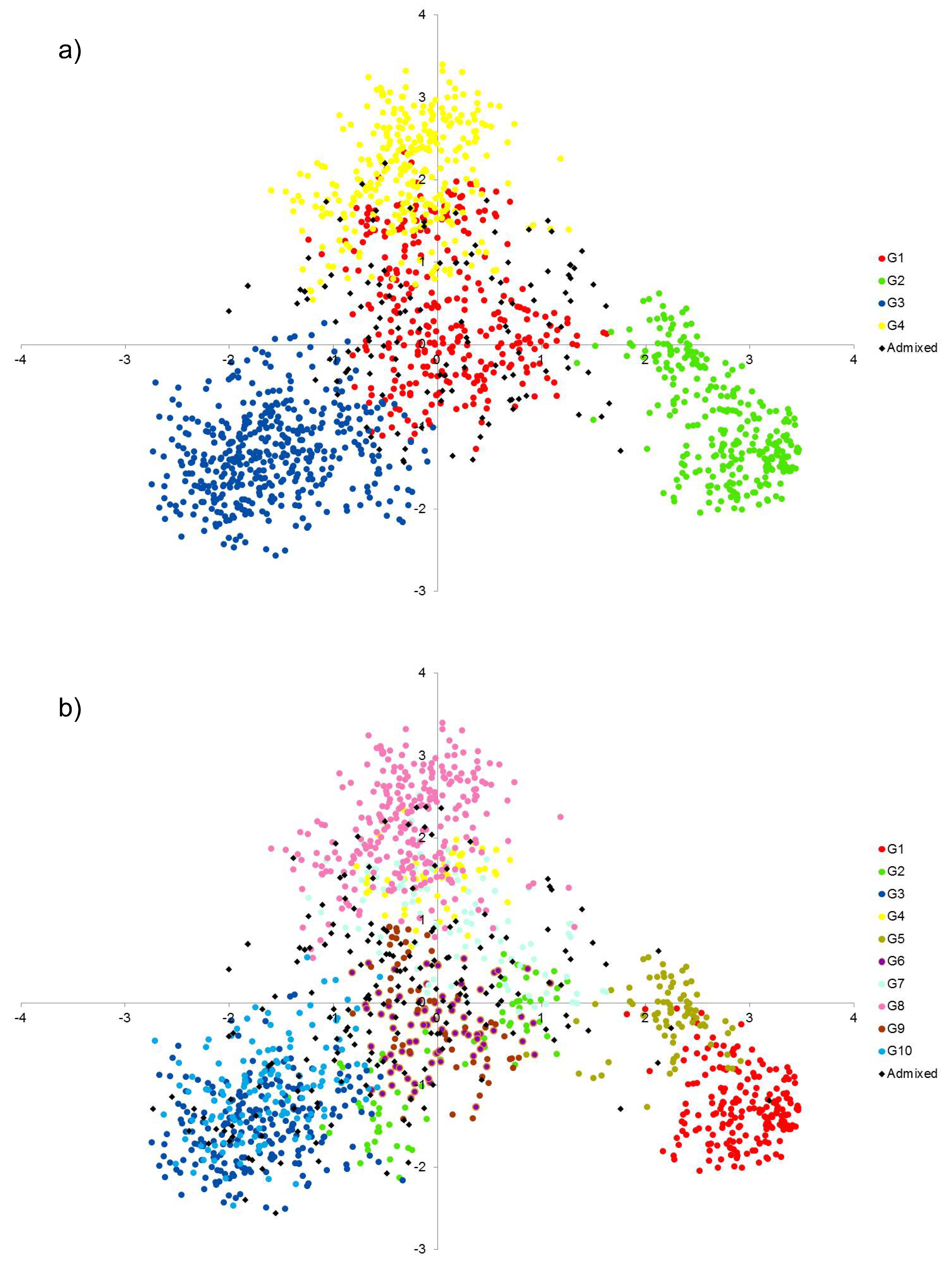

Supplement: S2 Fig — Scatter plot of 1485 barley landraces based on Principal Component Analysis calculated from 42 SSR data. a) for K = 4, b) for K = 10. Colours correspond to the different Structure inferred groups. (TIF) [file pone.0116164.s002.tif]

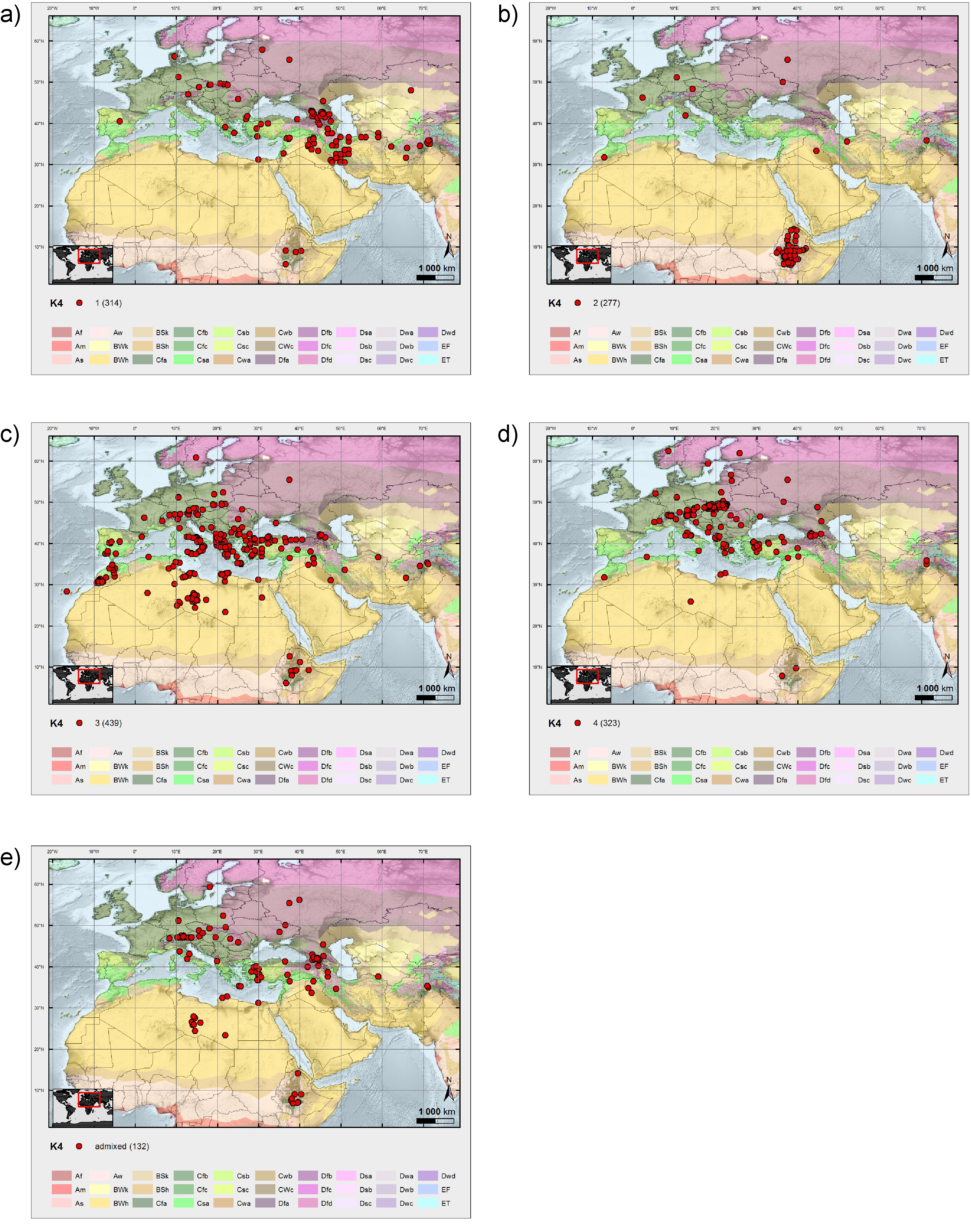

Supplement: S3 Fig — Geographical distribution of 1485 landraces over Köppen climate zones according to Structure inferred groups at K = 4. Each group (G1-G4) and admixed types were separately plotted. (a) G1; (b) G2; (c) G3; (d) G4; (e) admixed types. Climate abbreviations are explained in S6 Table. (TIF) [file pone.0116164.s003.tif]

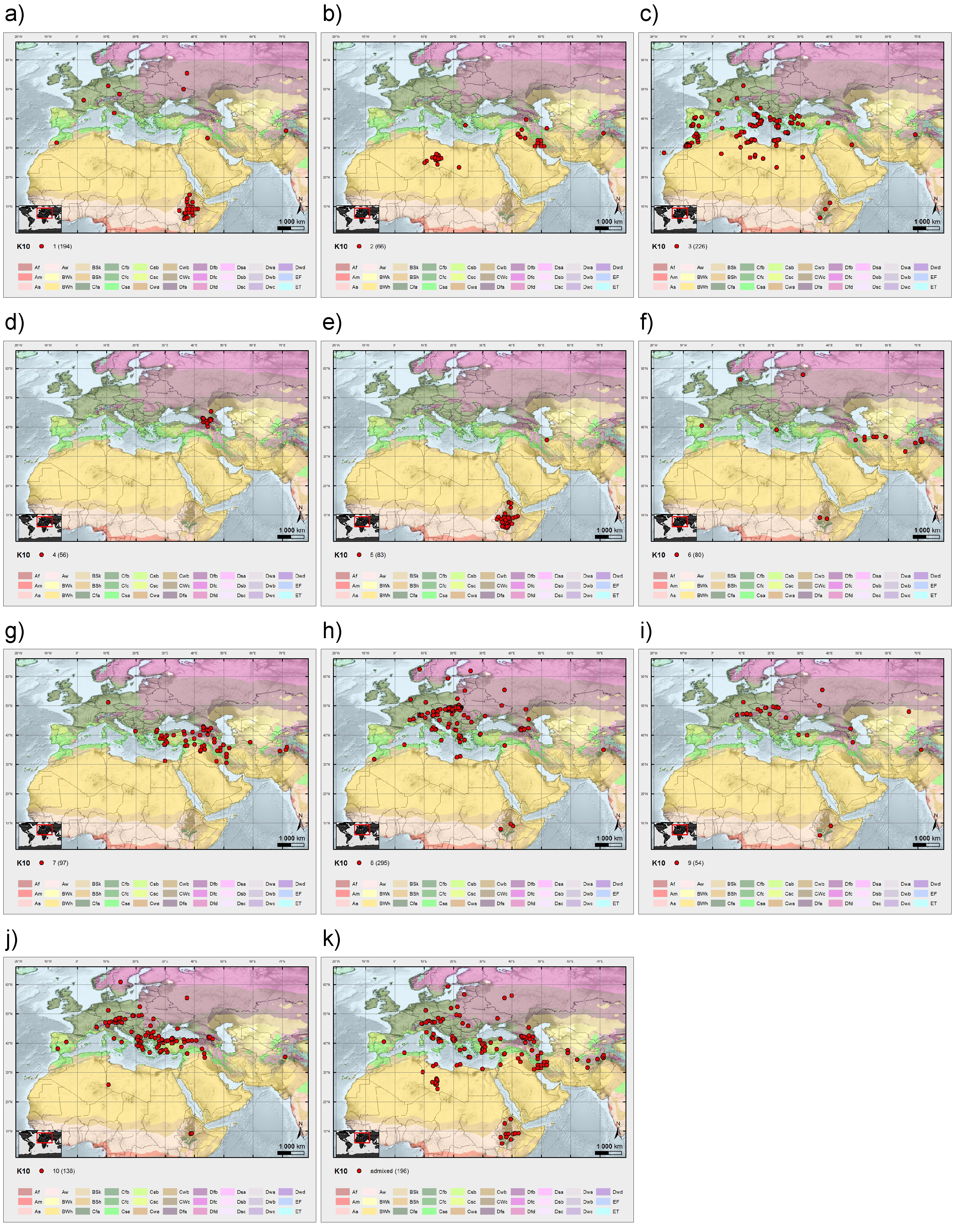

Supplement: S4 Fig — Geographical distribution of 1485 landraces over various Köppen climate zones according to Structure inferred groups at K = 10. Each group (G1-G10) and admixed types were separately plotted. (a) G1; (b) G2; (c) G3; (d) G4; (e) G5; (f) G6; (g) G7; (h) G8; (i) G9; (j) G10; (k) admixed types. Abbreviations are explained in S6 Table. (TIF) [file pone.0116164.s004.tif]

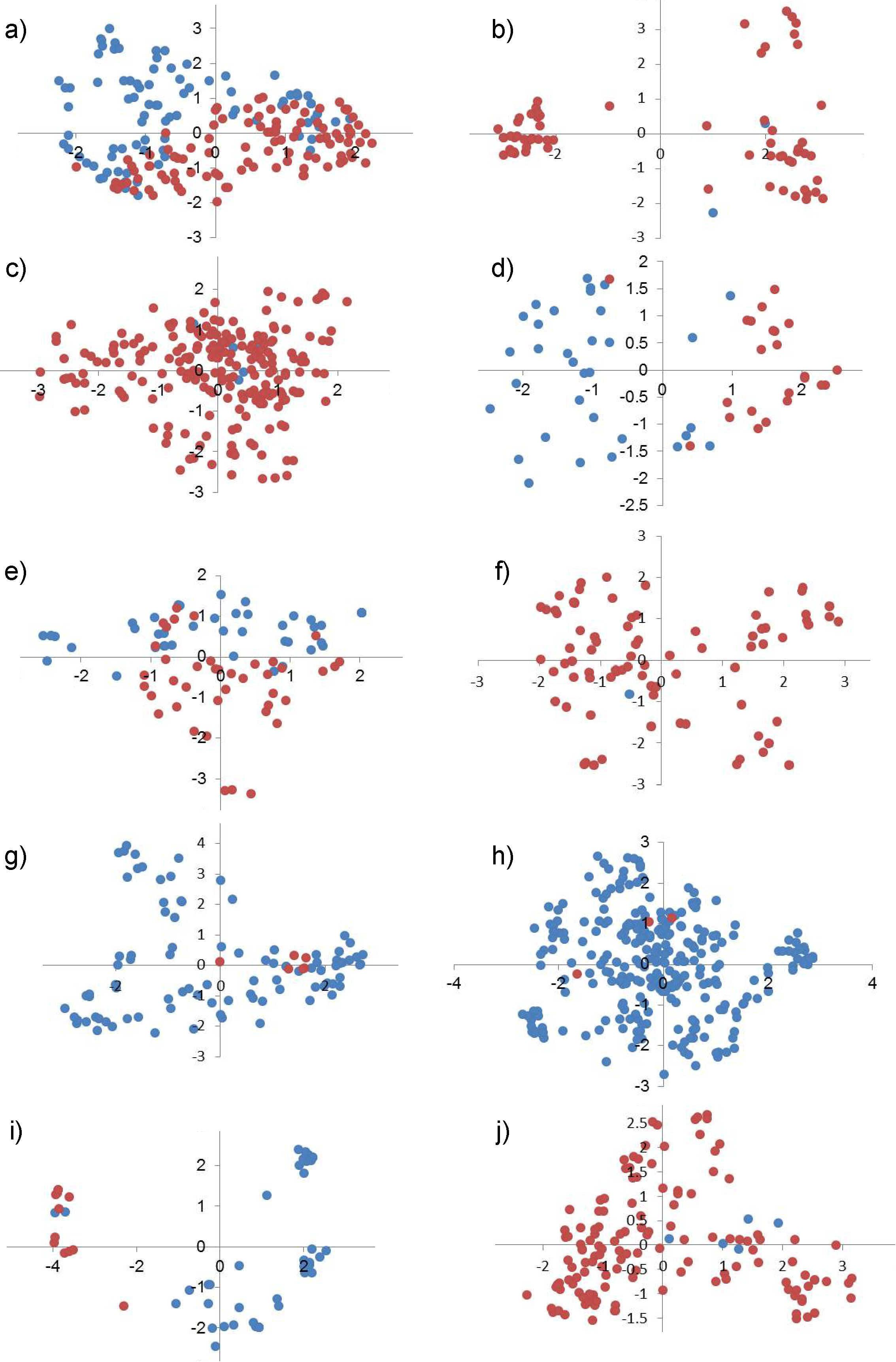

Supplement: S5 Fig — Individual PCA's for each Structure inferred group at K = 10. Each plot represents a single group: (a) G1, (b) G2, (c) G3, (d) G4, (e) G5, (f) G6, (g) G7, (h) G8, (i) G9, (j) G10. Blue circles indicate two-rowed and red circles six-rowed barleys. (TIF) [file pone.0116164.s005.tif]

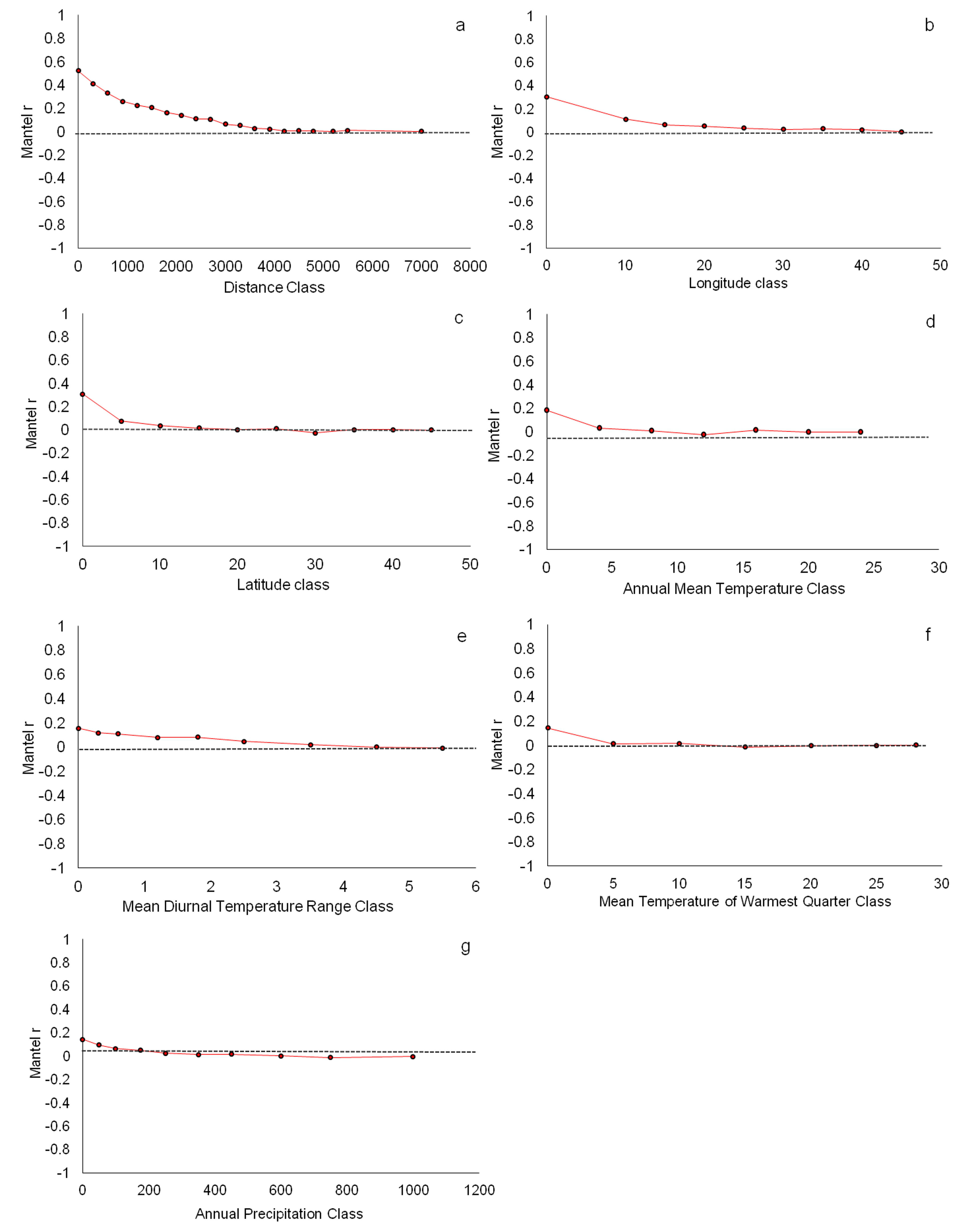

Supplement: S6 Fig — Correlograms showing spatial genetic autocorrelation patterns among: (a) genetic distance and geographical distance; (b) genetic distance and longitude; (c) genetic distance and latitude; (d) genetic distance and annual mean temperature; (e) genetic distance and mean diurnal range; (f) genetic distance and mean temperature of warmest quarter; (g) genetic distance and annual precipitation. The x-axis represents distinct classes and the y-axis represents the mantel r values. (TIF) [file pone.0116164.s006.tif]

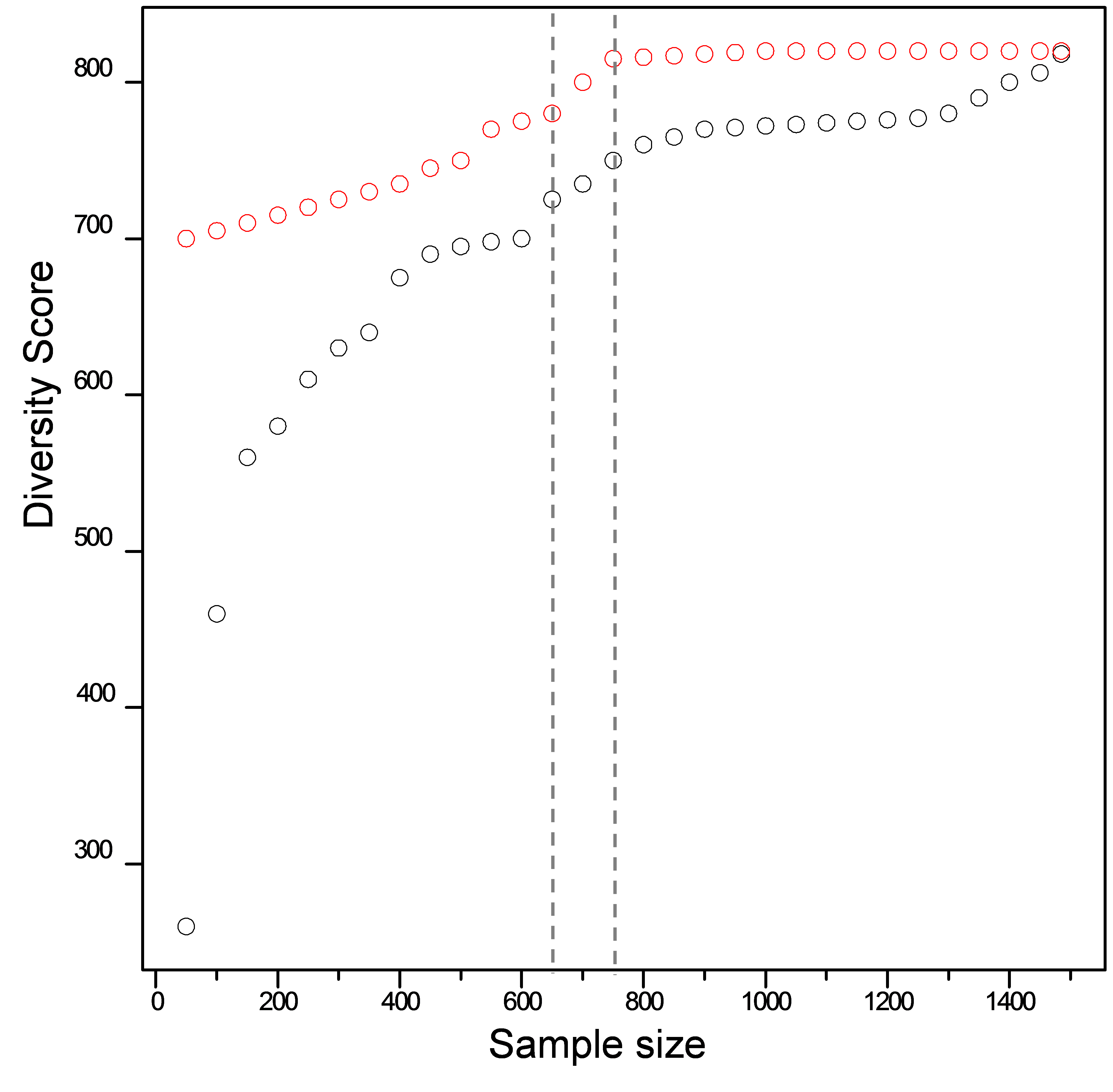

Supplement: S7 Fig — Comparing the sampling efficiency based on Mstrat and random sampling to capture most efficiently genetic diversity to establish a core reference set. Average diversity score calculated based on allelic richness was plotted against the sample size. Red circles indicate scores of the core collection using the M strategy and blue circles indicate scores of randomly selected accessions. (TIF) [file pone.0116164.s007.tif]

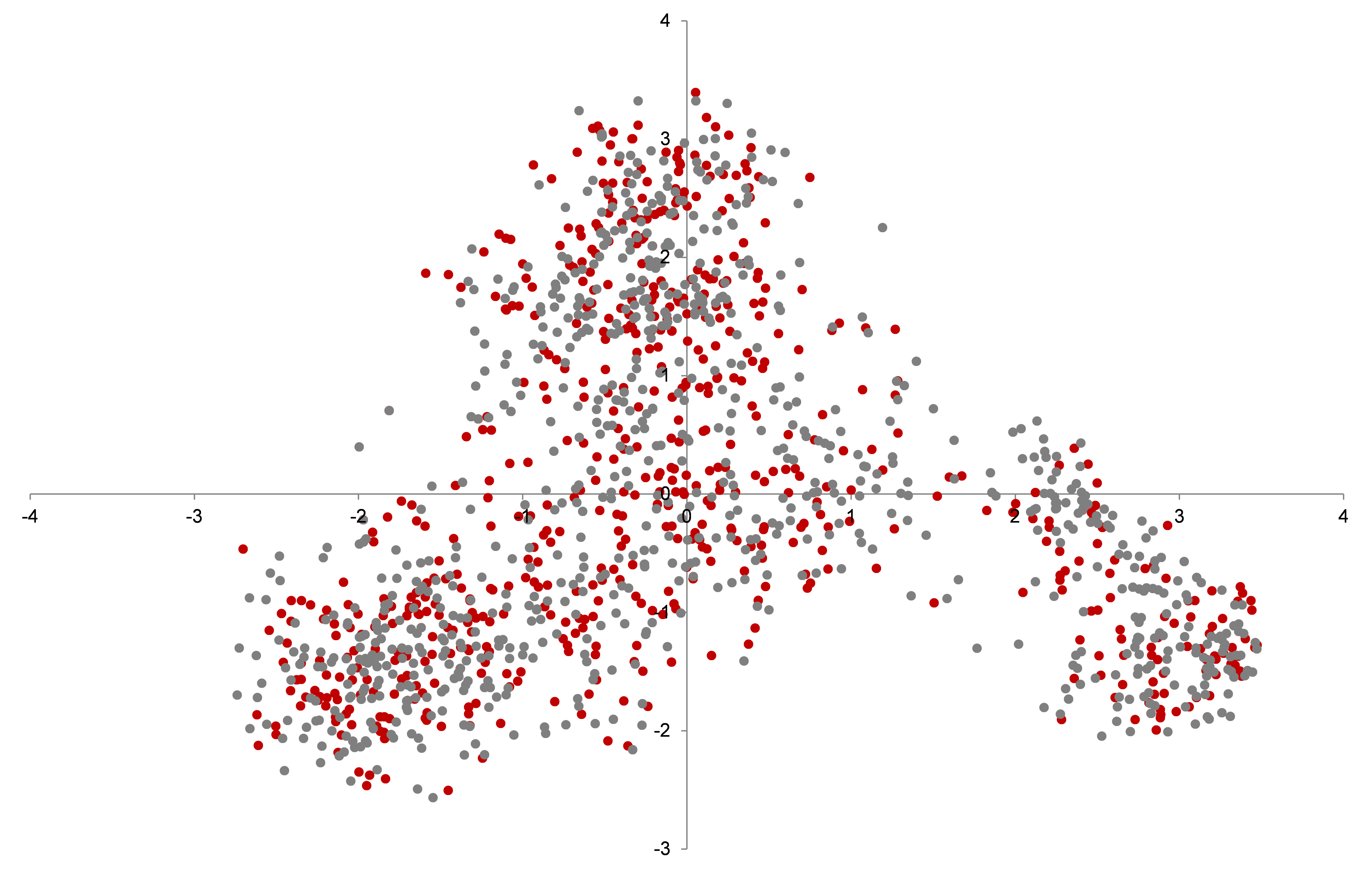

Supplement: S8 Fig — Scatter plot of Lrc1485 and Lrc648 based on PCA calculated from 42 SSR data. Landraces selected for Lrc648 are indicated in red colour. (TIF) [file pone.0116164.s008.tif]

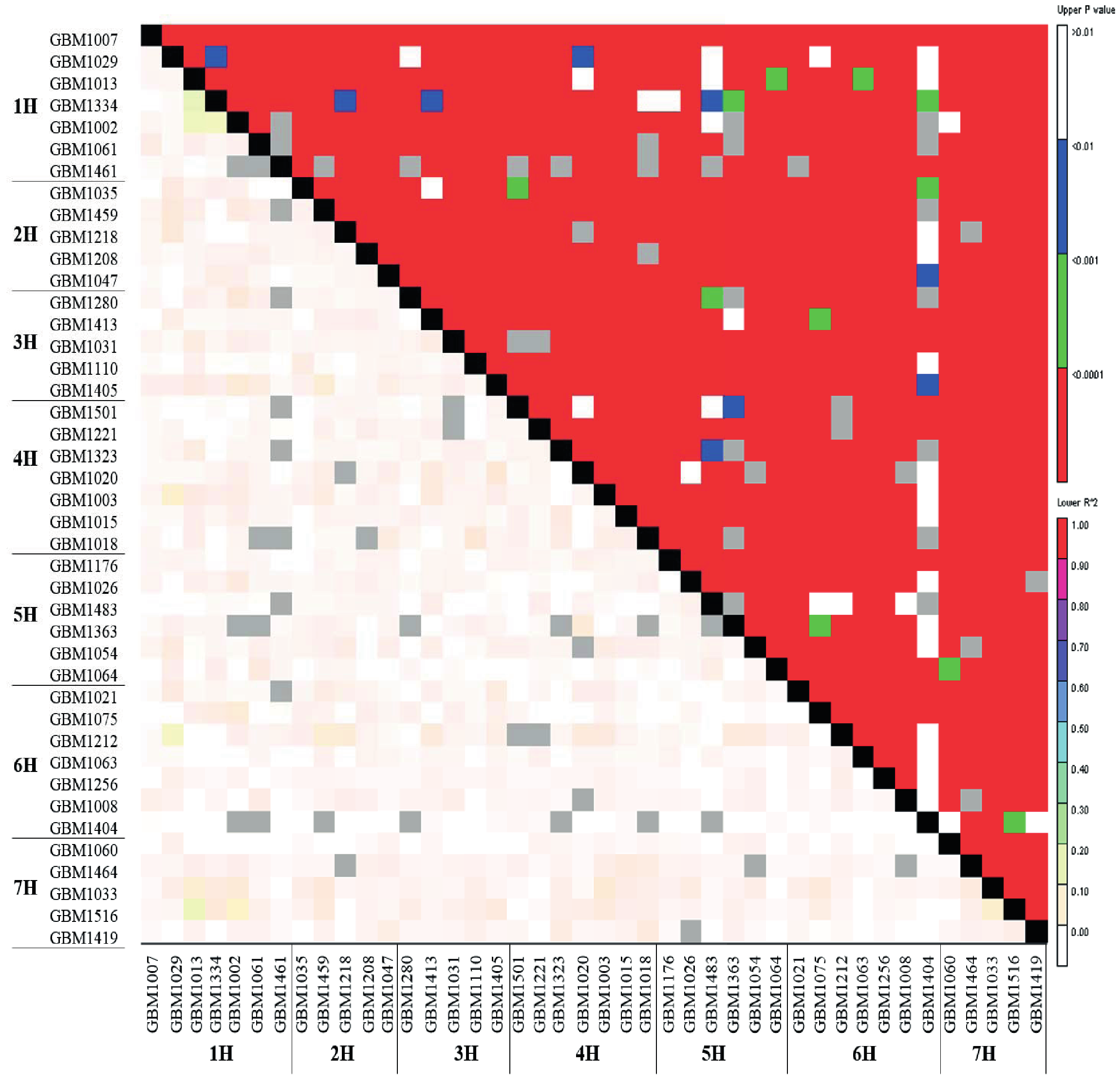

Supplement: S9 Fig — Linkage Disequilibrium (LD) display of 1485 barley landraces using 42 SSR markers. LD was calculated in TASSEL 2.1(www.maizegenetics.net/tassel) using 1000 permutations. Markers are arranged according to the genetic positions on barley genome (see S1 Fig.). (TIF) [file pone.0116164.s009.tif]

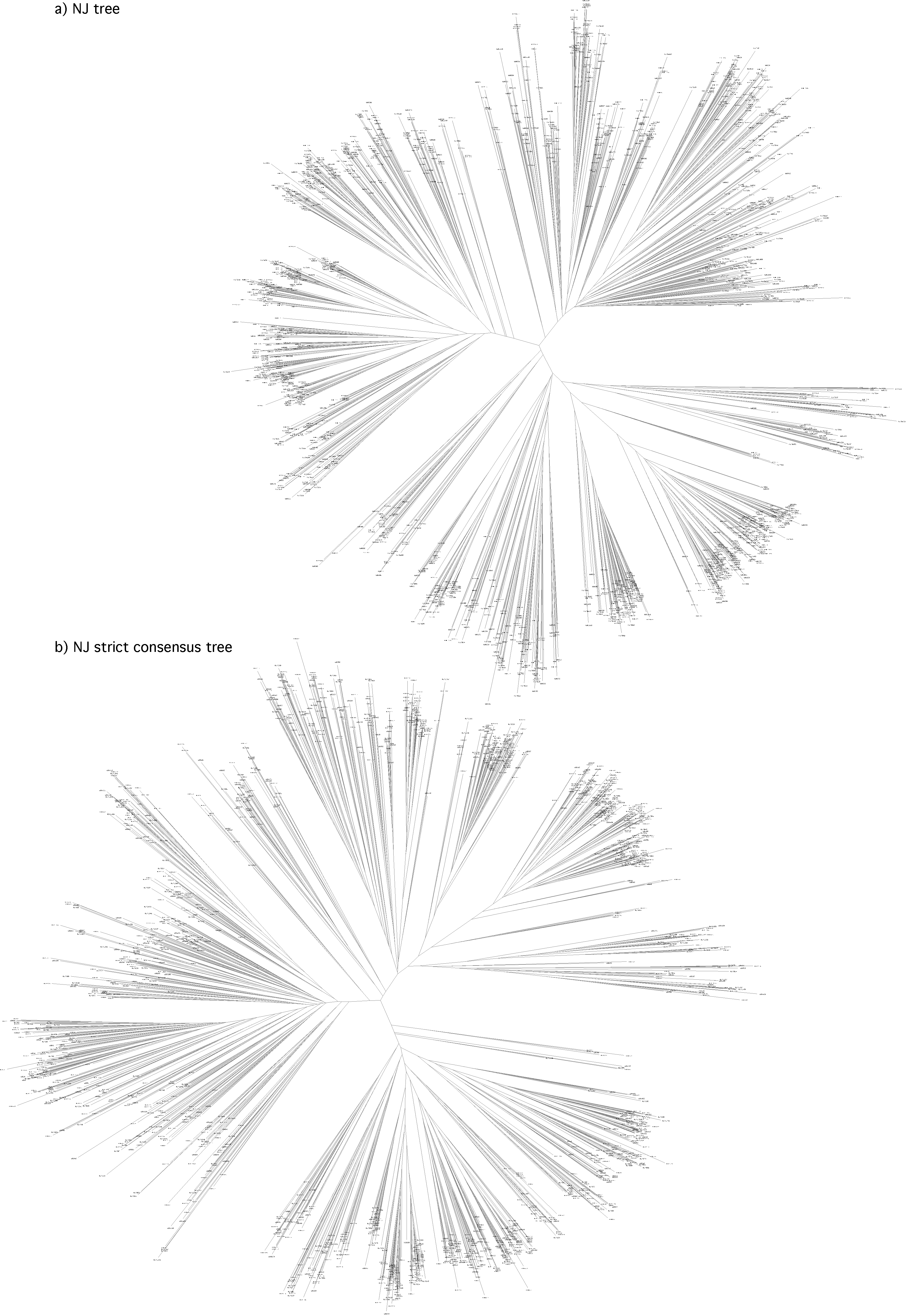

Supplement: S10 Fig — Evolutionary relationships of 1485 barley landraces I. The evolutionary history was inferred by a) a Neighbor-Joining tree, and b) a Neighbor-Joining strict consensus tree computed in SplitsTree software. (TIF) [file pone.0116164.s010.tif]

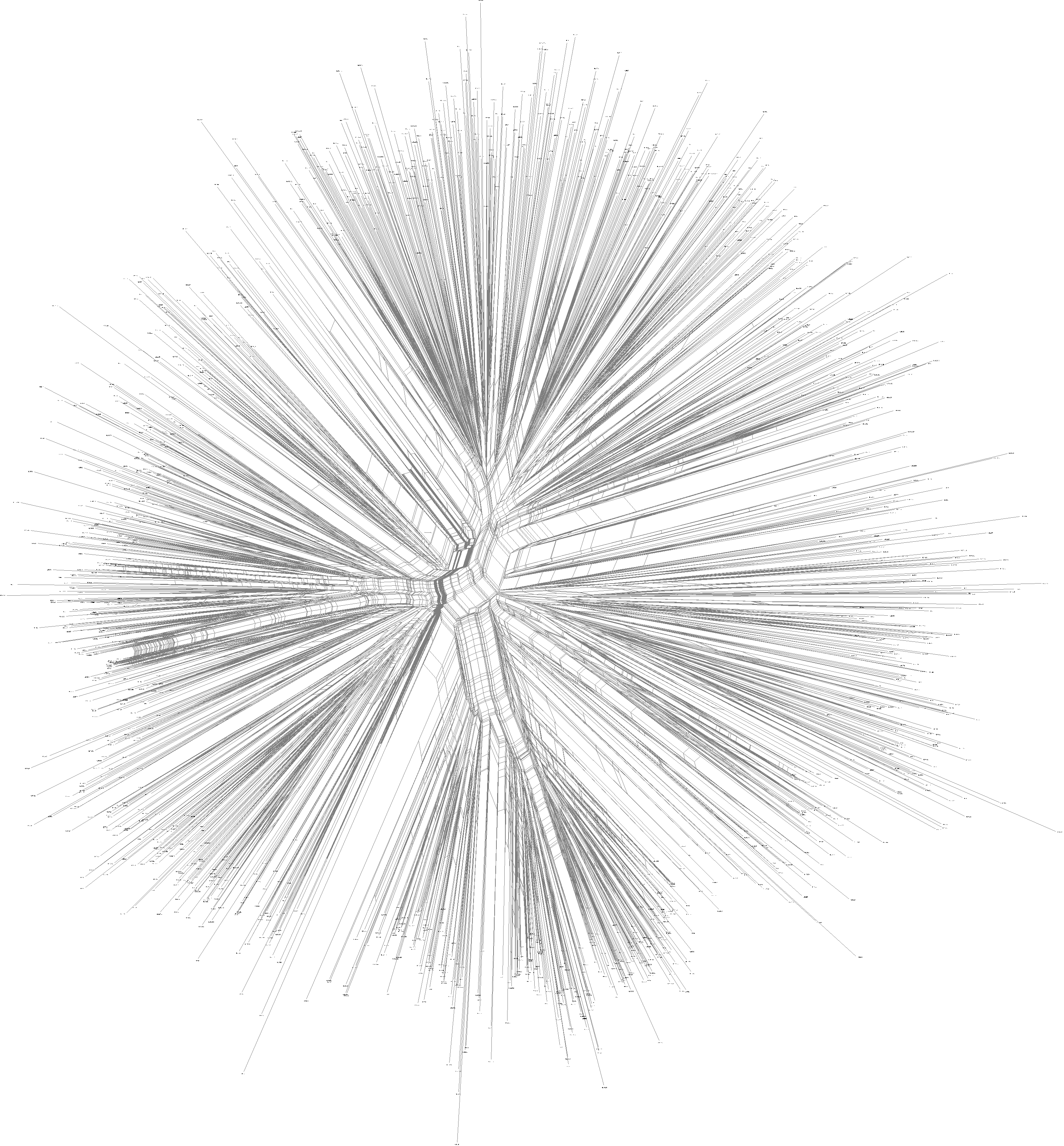

Supplement: S11 Fig — Evolutionary relationships of 1485 barley landraces II. The Neighbor-Net planar graph of uncorrected p-distances visualizes the high amount of reticulation in the collection (Taxa = 1485; Chars = 372; Fit = 90,615; Splits = 4604). (TIF) [file pone.0116164.s011.tif]
