# Supplementary material for: Genetic Diversity and Population Structure in a Legacy Collection of Spring Barley Landraces Adapted to a Wide Range of Climates
Source: PLoS One. 2014 Dec 26;9(12):e116164. doi: 10.1371/journal.pone.0116164 (PMC4277474; doi:10.1371/journal.pone.0116164)
Supplement: S3 Table — Pairwise comparison of Fst values between the Structure inferred groups groups a) for K = 4 and b) for K = 10. Significance of P-values computed after 1000 permutations are represented above diagonal and the Fst values are presented below. (DOCX) [file pone.0116164.s014.docx]

**Table S3.**

**a)**

| **Groups** | **G1** | **G2** | **G3** | **G4** |
| --- | --- | --- | --- | --- |
| **G1** | 0.000 | 0.001 | 0.001 | 0.001 |
| **G2** | 0.27906 | 0.000 | 0.001 | 0.001 |
| **G3** | 0.18699 | 0.34464 | 0.000 | 0.001 |
| **G4** | 0.20148 | **0.38782** | 0.26732 | 0.000 |

**b)**

| **Groups** | **G1** | **G2** | **G3** | **G4** | **G5** | **G6** | **G7** | **G8** | **G9** | **G10** |
| --- | --- | --- | --- | --- | --- | --- | --- | --- | --- | --- |
| **G1** | 0.000 | 0.001 | 0.001 | 0.001 | 0.001 | 0.001 | 0.001 | 0.001 | 0.001 | 0.001 |
| **G2** | 0.42393 | 0.000 | 0.001 | 0.001 | 0.001 | 0.001 | 0.001 | 0.001 | 0.001 | 0.001 |
| **G3** | 0.44377 | 0.28552 | 0.000 | 0.001 | 0.001 | 0.001 | 0.001 | 0.001 | 0.001 | 0.001 |
| **G4** | **0.55603** | 0.40965 | 0.39856 | 0.000 | 0.001 | 0.001 | 0.001 | 0.001 | 0.001 | 0.001 |
| **G5** | 0.35408 | 0.41626 | 0.43871 | 0.50713 | 0.000 | 0.001 | 0.001 | 0.001 | 0.001 | 0.001 |
| **G6** | 0.4525 | 0.26827 | 0.32977 | 0.39132 | 0.45582 | 0.000 | 0.001 | 0.001 | 0.001 | 0.001 |
| **G7** | 0.39019 | 0.26662 | 0.29139 | **0.19608** | 0.34937 | 0.25798 | 0.000 | 0.001 | 0.001 | 0.001 |
| **G8** | 0.45316 | 0.35282 | 0.33626 | 0.3398 | 0.39816 | 0.36719 | 0.22307 | 0.000 | 0.001 | 0.001 |
| **G9** | 0.49016 | 0.33954 | 0.36605 | 0.41481 | 0.51401 | 0.3273 | 0.29515 | 0.39375 | 0.000 | 0.01 |
| **G10** | 0.49639 | 0.34576 | 0.24733 | 0.42871 | 0.45858 | 0.34459 | 0.30306 | 0.37146 | 0.37311 | 0..000 |
