# Supplementary material for: Genetic Diversity and Population Structure in a Legacy Collection of Spring Barley Landraces Adapted to a Wide Range of Climates
Source: PLoS One. 2014 Dec 26;9(12):e116164. doi: 10.1371/journal.pone.0116164 (PMC4277474; doi:10.1371/journal.pone.0116164)
Supplement: S4 Table — Analysis of Molecular Variance (AMOVA). Summary of partitioning of genetic variation among and within different groups of Lrc1485. (DOCX) [file pone.0116164.s015.docx]

**Table S4.**

|  | Total | Among populations | Within populations | *P*-value* | F_st_ |
| --- | --- | --- | --- | --- | --- |
| **all Ethiopian *vs.* all non-Ethiopian** |  |  |  |  |  |
| Variance components | 13.428 | 2.652 | 10.776 | 0.000 | 0.197 |
| % variation | 100.000 | 19.750 | 80.250 | 0.000 |  |
| **all hulled *vs.* all naked** |  |  |  |  |  |
| Variance components | 13.087 | 2.116 | 10.971 | 0.000 | 0.162 |
| % variation | 100.000 | 16.170 | 83.830 | 0.000 |  |
| **all two-rowed *vs.* all six-rowed** |  |  |  |  |  |
| Variance components | 12.170 | 1.079 | 11.091 | 0.000 | 0.089 |
| % variation | 100.000 | 8.870 | 91.130 | 0.000 |  |
| **all hulled (non-Ethiopian) *vs.* all naked (non-Ethiopian)** |  |  |  |  |  |
| Variance components | 13.092 | 1.643 | 11.450 | 0.000 | 0.125 |
| % variation | 100.000 | 12.550 | 87.450 | 0.000 |  |
| **all hulled (Ethiopian) *vs.* all naked (Ethiopian)** |  |  |  |  |  |
| Variance components | 8.774 | 2.342 | 6.432 | 0.000 | 0.267 |
| % variation | 100.000 | 26.690 | 73.310 | 0.000 |  |
| **all Ethiopian hulled *vs.* total collection** |  |  |  |  |  |
| Variance components | 13.407 | 2.033 | 11.374 | 0.000 | 0.152 |
| % variation | 100.000 | 15.160 | 84.840 | 0.000 |  |
| **all Ethiopian hulled *vs.* all non-Ethiopian hulled** |  |  |  |  |  |
| Variance components | 13.740 | 2.542 | 11.198 | 0.000 | 0.185 |
| % variation | 100.000 | 18.500 | 81.500 | 0.000 |  |
| **all Ethiopian naked *vs.* all non-Ethiopian naked** |  |  |  |  |  |
| Variance components | 10.283 | 3.293 | 6.991 | 0.000 | 0.320 |
| % variation | 100.000 | 32.020 | 67.980 | 0.000 |  |
| **all two-rowed (non-Ethiopian) *vs.* all six-rowed (non-Ethiopian)** |  |  |  |  |  |
| Variance components | 12.469 | 1.587 | 10.882 | 0.000 |  |
| % variation | 100.000 | 12.730 | 87.270 | 0.000 | 0.127 |
| **all two-rowed (Ethiopian) *vs.* all six-rowed (Ethiopian)** |  |  |  |  |  |
| Variance components | 7.722 | 7.229 | 0.492 | 0.000 |  |
| % variation | 100.000 | **6.380** | **93.620** | 0.000 | 0.064 |
| **all two-rowed (Ethiopian) *vs.* all two-rowed (non-Ethiopian)** |  |  |  |  |  |
| Variance components | 12.231 | 2.773 | 9.459 | 0.000 | 0.227 |
| % variation | 100.000 | 22.670 | 77.330 | 0.000 |  |
| **all six-rowed (Ethiopian) *vs.* all six-rowed (non-Ethiopian)** |  |  |  |  |  |
| Variance components | 14.411 | 3.424 | 10.987 | 0.000 |  |
| % variation | 100.000 | 23.760 | 76.240 | 0.000 | 0.238 |
| **Structure inferred groups (*K* *=* 4)** |  |  |  |  |  |
| Variance components | 12.506 | 3.418 | 9.088 | 0.000 |  |
| % variation | 100.000 | 27.330 | 72.670 | 0.000 | 0.233 |
| **Structure inferred groups (*K* *=* 10)** |  |  |  |  |  |
| Variance components | 12.164 | 4.573 | 7.591 | 0.000 |  |
| % variation | 100.000 | **37.600** | **62.400** | 0.000 | 0.375 |
