# Supplementary material for: Genetic Diversity and Population Structure in a Legacy Collection of Spring Barley Landraces Adapted to a Wide Range of Climates
Source: PLoS One. 2014 Dec 26;9(12):e116164. doi: 10.1371/journal.pone.0116164 (PMC4277474; doi:10.1371/journal.pone.0116164)
Supplement: S5 Table — Mantel correlogram tables. a) Mantel correlogram tables between: genetic distance and geographic distance; b) genetic distance and longitude difference matrix; c) genetic distance and latitude difference matrix; d) genetic distance and annual mean temperature; e) genetic distance and mean diurnal range; f) genetic distance and temperature of warmest quarter; and g) genetic distance and annual precipitation. 1different distance classes are shown; 2lower and upper boundary values for each class; 3number of pairs for which the correlation was calculated within each distance class; 4the mantle correlation for each class; 5the significance of mantel correlation for each class. (DOCX) [file pone.0116164.s016.docx]

**Table S5.**

**Table S3a** Mantel correlogram between *genetic distance and geographic distance*. ^*^in kilometer

| Class^1^ | Min^2*^ | Max^2*^ | Pairs^3^ | Mantel r^4^ | P-value^5^ |
| --- | --- | --- | --- | --- | --- |
| 1 | 0 | 300 | 73503 | 0.52327 | 0.002 |
| 2 | 300 | 600 | 51623 | 0.4131 | 0.002 |
| 3 | 600 | 900 | 54160 | 0.32875 | 0.002 |
| 4 | 900 | 1200 | 55200 | 0.25814 | 0.002 |
| 5 | 1200 | 1500 | 52360 | 0.2235 | 0.002 |
| 6 | 1500 | 1800 | 48593 | 0.20506 | 0.002 |
| 7 | 1800 | 2100 | 73270 | 0.16337 | 0.002 |
| 8 | 2100 | 2400 | 59780 | 0.14068 | 0.002 |
| 9 | 2400 | 2700 | 67085 | 0.10775 | 0.002 |
| 10 | 2700 | 3000 | 55063 | 0.10537 | 0.002 |
| 11 | 3000 | 3300 | 74808 | 0.06373 | 0.002 |
| 12 | 3300 | 3600 | 56716 | 0.05144 | 0.002 |
| 13 | 3600 | 3900 | 64816 | 0.02578 | 0.002 |
| 14 | 3900 | 4200 | 43833 | 0.02011 | 0.002 |
| 15 | 4200 | 4500 | 69709 | 0.00342 | 0.03393 |
| 16 | 4500 | 4800 | 53184 | 0.0064 | 0.002 |
| 17 | 4800 | 5200 | 86547 | 0.00311 | 0.03992 |
| 18 | 5200 | 5500 | 32814 | 0.001 | 0.17166 |
| 19 | 5500 | 7000 | 32051 | 0.00969 | 0.002 |
| 20 | 7000 | 8000 | 4249 | 0.0019 | 0.002 |

**Table S3b.** Mantel correlogram between *genetic distance and longitude* difference matrix. ^*^in degree (°)

| Class^1^ | Min^2*^ | Max^2*^ | Pairs^3^ | Mantel r^4^ | P-value^5^ |
| --- | --- | --- | --- | --- | --- |
| 1 | 0 | 10 | 372933 | 0.30142 | 0.000 |
| 2 | 10 | 15 | 99706 | 0.11062 | 0.000 |
| 3 | 15 | 20 | 133628 | 0.06165 | 0.000 |
| 4 | 20 | 25 | 131885 | 0.04863 | 0.000 |
| 5 | 25 | 30 | 124302 | 0.03342 | 0.000 |
| 6 | 30 | 35 | 78835 | 0.02128 | 0.000 |
| 7 | 35 | 40 | 28578 | 0.02702 | 0.000 |
| 8 | 40 | 45 | 25651 | 0.01875 | 0.000 |
| 9 | 45 | 50 | 41363 | 0.00026 | 0.000 |

**Table S3c.** Mantel correlogram between *genetic distance and latitude* difference matrix. ^*^in degree (°)

| Class^1^ | Min2^*^ | Max^2*^ | Pairs^3^ | Mantel r^4^ | P-value^5^ |
| --- | --- | --- | --- | --- | --- |
| 1 | 0 | 5 | 297005 | 0.30807 | 0.001 |
| 2 | 5 | 10 | 203938 | 0.07606 | 0.001 |
| 3 | 10 | 15 | 150791 | 0.03572 | 0.001 |
| 4 | 15 | 20 | 92041 | 0.01649 | 0.001 |
| 5 | 20 | 25 | 73157 | 0.002 | 0.045 |
| 6 | 25 | 30 | 90996 | 0.01077 | 0.041 |
| 7 | 30 | 35 | 77656 | -0.02792 | 0.011 |
| 8 | 35 | 40 | 58811 | 0.00204 | 0.096 |
| 9 | 40 | 45 | 61506 | 0.00296 | 0.026 |
| 10 | 45 | 65 | 4894 | -0.00026 | 0.473 |

**Table S3d.** Mantel correlogram between *genetic distance and annual mean temperature (AMT)* difference matrix. ^*^in °C

| Class^1^ | Min^2*^ | Max^2*^ | Pairs^3^ | Mantel r^4^ | P-value^5^ |
| --- | --- | --- | --- | --- | --- |
| 1 | 0 | 4 | 367291 | 0.18153 | 0.001 |
| 2 | 4 | 8 | 300352 | 0.0327 | 0.001 |
| 3 | 8 | 12 | 225124 | 0.00794 | 0.001 |
| 4 | 12 | 16 | 129637 | -0.02379 | 0.001 |
| 5 | 16 | 20 | 62951 | 0.01253 | 0.001 |
| 6 | 20 | 24 | 20489 | -0.00525 | 0.001 |
| 7 | 24 | 28 | 4951 | -0.00109 | 0.001 |

**Table S3e.** Mantel correlogram between *genetic distance and mean diurnal range (MDR)* difference matrix. ^*^in °C

| Class^1^ | Min^2*^ | Max^2*^ | Pairs^3^ | Mantel r^4^ | P-value^5^ |
| --- | --- | --- | --- | --- | --- |
| 1 | 0 | 0.3 | 92152 | 0.15405 | 0.001 |
| 2 | 0.3 | 0.6 | 76932 | 0.11576 | 0.001 |
| 3 | 0.6 | 1.2 | 127131 | 0.10915 | 0.001 |
| 4 | 1.2 | 1.8 | 118454 | 0.07773 | 0.001 |
| 5 | 1.8 | 2.5 | 122936 | 0.08152 | 0.001 |
| 6 | 2.5 | 3.5 | 126397 | 0.04573 | 0.001 |
| 7 | 3.5 | 4.5 | 133784 | 0.01896 | 0.001 |
| 8 | 4.5 | 5.5 | 113506 | 0.00025 | 0.876 |
| 9 | 5.5 | 6.5 | 106984 | -0.00941 | 0.081 |

**Table S3f.** Mantel correlogram between *genetic distance and mean temperature of warmest quarter (MTW)* difference matrix. ^*^in °C

| Class^1^ | Min^2*^ | Max^*^ | Pairs^3^ | Mantel r^4^ | P-value^5^ |
| --- | --- | --- | --- | --- | --- |
| 1 | -5 | 5 | 536095 | 0.14177 | 0.001 |
| 2 | 5 | 10 | 332327 | 0.01248 | 0.001 |
| 3 | 10 | 15 | 164292 | 0.01382 | 0.001 |
| 4 | 15 | 20 | 63174 | -0.01488 | 0.001 |
| 5 | 20 | 25 | 13556 | -0.00465 | 0.041 |
| 6 | 25 | 28 | 1121 | -0.00023 | 0.002 |
| 7 | 28 | 30 | 230 | -0.00008 | 0.008 |

**Table S3g.** Mantel correlogram between genetic distance and annual mean precipitation (APT) difference matrix. ^2^in millimeter (mm)

| Class^1^ | Min^2^ | Max^2^ | Pairs^3^ | Mantel r^4^ | P-value^5^ |
| --- | --- | --- | --- | --- | --- |
| 1 | 0 | 50 | 90136 | 0.14122 | 0.001 |
| 2 | 50 | 100 | 84094 | 0.09324 | 0.001 |
| 3 | 100 | 175 | 120210 | 0.06312 | 0.001 |
| 4 | 175 | 250 | 121581 | 0.04899 | 0.001 |
| 5 | 250 | 350 | 145333 | 0.02449 | 0.001 |
| 6 | 350 | 450 | 124786 | 0.01413 | 0.001 |
| 7 | 450 | 600 | 152274 | 0.01497 | 0.001 |
| 8 | 600 | 750 | 107351 | 0.00268 | 0.018 |
| 9 | 750 | 1000 | 103732 | -0.0134 | 0.001 |
| 10 | 1000 | 1500 | 54523 | -0.0047 | 0.001 |
