# Supplementary material for: Genetic Diversity and Population Structure in a Legacy Collection of Spring Barley Landraces Adapted to a Wide Range of Climates
Source: PLoS One. 2014 Dec 26;9(12):e116164. doi: 10.1371/journal.pone.0116164 (PMC4277474; doi:10.1371/journal.pone.0116164)
Supplement: S7 Table — Comparison of diversity statistics for different sample sizes of core groups generated from 1485 accessions as well as Lrc1485, Lrc1014, Lrc648 and Lrc648r using 42 SSR markers and climatic variables. N - number of accessions; AN - average allele number; GD - gene diversity; PIC - polymorphism information content; MAF - average major allele frequency. (DOCX) [file pone.0116164.s018.docx]

**Table S7.**

|  |  |  | |  |  | Group size | | | | | | |
| --- | --- | --- | --- | --- | --- | --- | --- | --- | --- | --- | --- | --- |
| N | 200 | | 400 | 600 | **Lrc648** | | **Lrc648r** | 745 | 800 | 1000 | **Lrc1014** | **Lrc1485** |
| AN | 8.17 | | 8.21 | 8.29 | 8.38 | | 7.54 | 8.50 | 8.86 | 8.86 | 8.89 | 8.95 |
| GD | 0.61 | | 0.61 | 0.61 | 0.60 | | 0.59 | 0.60 | 0.61 | 0.61 | 0.60 | 0.60 |
| PIC | 0.56 | | 0.55 | 0.55 | 0.55 | | 0.54 | 0.55 | 0.55 | 0.55 | 0.55 | 0.55 |
| MAF | 0.51 | | 0.51 | 0.51 | 0.51 | | 0.51 | 0.52 | 0.51 | 0.51 | 0.51 | 0.51 |
