# Supplementary material for: Genetic Diversity and Population Structure in a Legacy Collection of Spring Barley Landraces Adapted to a Wide Range of Climates
Source: PLoS One. 2014 Dec 26;9(12):e116164. doi: 10.1371/journal.pone.0116164 (PMC4277474; doi:10.1371/journal.pone.0116164)
Supplement: S8 Table — Comparison of phenotypic diversity between the core set Lrc648 (based on Mstrat) and the random set Lrc648r. Min. - minimum; Max. – maximum; SD. - standard deviation of the measured traits heading date (Hd) (in days to flowering), spike length (Sl) (in cm) and plant height (Ht) (cm). (DOCX) [file pone.0116164.s019.docx]

**Table S8** **Comparison of phenotypic diversity between the core set Lrc648 (based on Mstrat) and the random set Lrc648r of landrace accessions.** Min. - minimum; Max. – maximum; SD. - standard deviation of the measured traits heading date (Hd) (in days to flowering), spike length (Sl) (in cm) and plant height (Ht) (cm).

|  | **Hd_ Lrc648** | **Hd_ Lrc648r** | **Sl_ Lrc648** | **Sl_ Lrc648r** | **Ht_ Lrc648** | **Ht_ Lrc648r** |
| --- | --- | --- | --- | --- | --- | --- |
| Min. | 49 | 51 | 3 | 2.75 | 38 | 42 |
| Max | 75 | 71 | 13.5 | 13.25 | 170 | 120 |
| SD. | 4.56 | 3.64 | 2.20 | 2.36 | 15.63 | 16.45 |
